# Metabolic Complementarity and Genomics of the Dual Bacterial Symbiosis of Sharpshooters

**DOI:** 10.1371/journal.pbio.0040188

**Published:** 2006-06-06

**Authors:** Dongying Wu, Sean C Daugherty, Susan E Van Aken, Grace H Pai, Kisha L Watkins, Hoda Khouri, Luke J Tallon, Jennifer M Zaborsky, Helen E Dunbar, Phat L Tran, Nancy A Moran, Jonathan A Eisen

**Affiliations:** **1**The Institute for Genomic Research, Rockville, Maryland, United States of America; **2**J. Craig Venter Institute, Joint Technology Center, Rockville, Maryland, United States of America; **3**Department of Ecology and Evolutionary Biology, University of Arizona, Tucson, Arizona, United States of America; The Sanger InstituteUnited Kingdom

## Abstract

Mutualistic intracellular symbiosis between bacteria and insects is a widespread phenomenon that has contributed to the global success of insects. The symbionts, by provisioning nutrients lacking from diets, allow various insects to occupy or dominate ecological niches that might otherwise be unavailable. One such insect is the glassy-winged sharpshooter *(Homalodisca coagulata),* which feeds on xylem fluid, a diet exceptionally poor in organic nutrients. Phylogenetic studies based on rRNA have shown two types of bacterial symbionts to be coevolving with sharpshooters: the gamma-proteobacterium Baumannia cicadellinicola and the *Bacteroidetes* species Sulcia muelleri. We report here the sequencing and analysis of the 686,192–base pair genome of B. cicadellinicola and approximately 150 kilobase pairs of the small genome of *S. muelleri,* both isolated from *H. coagulata.* Our study, which to our knowledge is the first genomic analysis of an obligate symbiosis involving multiple partners, suggests striking complementarity in the biosynthetic capabilities of the two symbionts: B. cicadellinicola devotes a substantial portion of its genome to the biosynthesis of vitamins and cofactors required by animals and lacks most amino acid biosynthetic pathways, whereas S. muelleri apparently produces most or all of the essential amino acids needed by its host. This finding, along with other results of our genome analysis, suggests the existence of metabolic codependency among the two unrelated endosymbionts and their insect host. This dual symbiosis provides a model case for studying correlated genome evolution and genome reduction involving multiple organisms in an intimate, obligate mutualistic relationship. In addition, our analysis provides insight for the first time into the differences in symbionts between insects (e.g., aphids) that feed on phloem versus those like H. coagulata that feed on xylem. Finally, the genomes of these two symbionts provide potential targets for controlling plant pathogens such as *Xylella fastidiosa,* a major agroeconomic problem, for which H. coagulata and other sharpshooters serve as vectors of transmission.

## Abstract 2

Sequence data from two obligate bacterial endosymbionts of an insect--the glassy-winged sharpshooter--suggest there is metabolic co-dependency among them and their insect host.

## Introduction

Through mutualistic symbioses with bacteria, eukaryotes have been able to acquire metabolic capabilities that in turn have allowed the utilization of otherwise unavailable ecological niches. Among the diverse examples of such symbioses, those involving bacteria that live inside the cells of their host are of great interest. These “endo”-symbioses played a central role in the early evolution of eukaryotes (e.g., the establishment of the mitochondria and chloroplasts) and in many more recent diversification events such as animals living at deep-sea vents, corals, blood-feeding flies, carpenter ants, and several clades of sap-feeding insects.

Insects that feed primarily or entirely on sap are a virtual breeding ground for symbioses because this liquid rarely contains sufficient quantities of the nutrients that animals are unable to make for themselves. For example, the sole diet of most aphids is sap from phloem which is the component of the plant vascular system normally used to transport sugars and other organic nutrients. Despite the presence of many nutrients, phloem usually has little, if any, of the “essential” amino acids that cannot be synthesized by animals. To compensate, aphids engage in an obligate symbiosis with bacteria in the genus *Buchnera,* which, in exchange for sugar and simple, nonessential amino acids, synthesize the needed essential amino acids for their hosts.

The exact details of aphid- *Buchnera* interactions have been difficult to determine because no *Buchnera* has been cultivated outside its host. This limitation has been circumvented to a large degree by sequencing and analysis of multiple *Buchnera* genomes [ 1– 3], which have provided detailed insights into the biology, evolution, and ecology of these symbioses. For example, despite having undergone massive amounts of gene loss in the time after they diverged from free-living *Gammaproteobacteria,* the *Buchnera* encode many pathways for the synthesis of essential amino acids. A critical component of these genomic studies is that, in most aphids, *Buchnera* is the only symbiont [ 4]. This implies that when genome-based metabolic pathway reconstructions suggest that a particular *Buchnera* is unable to make all the essential nutrients for its host, either the reconstructions are wrong, or the host must be getting those nutrients from its diet. For example, although one of the *Buchnera* strains is predicted to not be able to incorporate inorganic sulfur for the production of cysteine and other compounds, sulfur-containing organic compounds are known to occur in the diet of its host aphid [ 2].

In many other sap-feeding insects, including some aphids, several heritable bacterial types are found often living in close proximity within specialized structures in the insect body (e.g., [ 5– 9]). This is apparently the case for all insects that are strict xylem-sap feeders, which include cicadas, spittlebugs, and some leafhoppers [ 5]. Xylem is the component of the plant vascular system that is primarily used to transport water and salts from the roots to the rest of the plant. Xylem sap has the lowest nitrogen or carbon content of any plant component and contains few organic compounds [ 10]. Although the composition varies among plant species and developmental stages, xylem fluid is always nutrient-poor, containing mostly inorganic compounds and minerals with small amounts of amino acids and organic acids [ 11– 15]. As in phloem, the amino acids consist mainly of nonessential types such as glutamine, asparagine, and aspartic acid, with all essential ones absent or present in very low amounts.

Among xylem-feeders, sharpshooters (Insecta: Hemiptera: Cicadellidae: Cicadellinae) are a prominent group of about 2,000 species [ 10], many of which are major pests of agriculture due to their roles as vectors of plant pathogens. Sharpshooters are known to possess two bacterial symbionts. One, called Candidatus Baumannia cicadellinicola (hereafter *Baumannia*), resembles *Buchnera* in having small genome size and a biased nucleotide composition favoring adenine and thymine (A + T) and in belonging to the Enterobacteriales group in the *Gammaproteobacteria* [ 16]. The other symbiont, which was recently named Candidatus Sulcia muelleri (hereafter *Sulcia*), is in the *Bacteroidetes* phylum (formerly called the Cytophaga-Flexibacter-Bacteroides, or CFB, phylum) and is distributed widely in related insect hosts [ 9]. Both symbionts are vertically transmitted in eggs and are housed in a specialized bacteriome within developing nymphs and adults, and molecular phylogenetic studies show that both symbionts represent ancient associations dating to the origin of sharpshooters *(Baumannia)* or earlier *(Sulcia)* [ 5, 9, 16].

We sought to apply genome sequencing and analysis methods to the sharpshooter symbioses. For a host species, we selected the glassy-winged sharpshooter, Homalodisca coagulata. This pest species has a rapidly expanding geographic range and inflicts major crop damage as a vector for the bacterium *Xylella fastidiosa,* the agent of Pierce's disease of grapes and other plant diseases [ 10]. Initially, we focused on the *Baumannia* symbiont with the idea that comparisons with the related *Buchnera* species would allow us to better identify differences that related to xylem versus phloem feeding. After completing the genome of this *Baumannia,* analysis revealed that many pathways that we expected to be present were missing. In contrast to the *Buchnera-*aphid symbioses, a second symbiont is present in sharpshooters, so we could not assume that the nutrients that would have been made by the missing pathways must be in the sharpshooter diet. Despite technical difficulties, we were able to obtain a significant portion of the genome of the *Sulcia* from the same wild-caught samples of *H. coagulata.*

Here we present the analysis of these two genomic datasets and the striking finding that the symbionts appear to work in concert, and possibly even share metabolites, to produce all of the nutrients needed by the host to survive on its xylem diet.

## Results/Discussion

### General Features of the *Baumannia* Genome and Predicted Genes

The genome of *Baumannia* consists of one circular chromosome of 686,192 base pairs (bp) with an average G + C content of 33.23% ( Table 1). The genome size closely matches an earlier estimate from gel electrophoresis [ 16]. *Baumannia* has neither a strong GC skew pattern nor a *dnaA* homolog—two features commonly used to identify origins of replication in bacteria. A putative origin was identified and designated as position 1, based on a weak but clear transition in oligonucleotide skew.

**Table 1:**
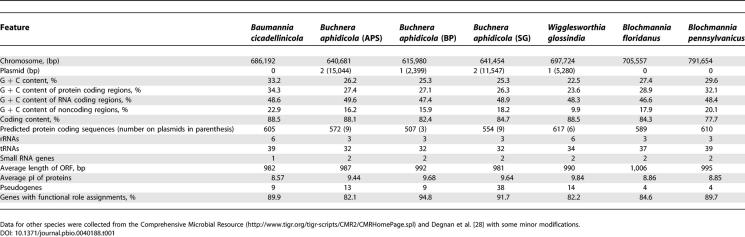
General Features of the Genomes of *Baumannia* and Other Insect Endosymbionts

**Figure 1:**
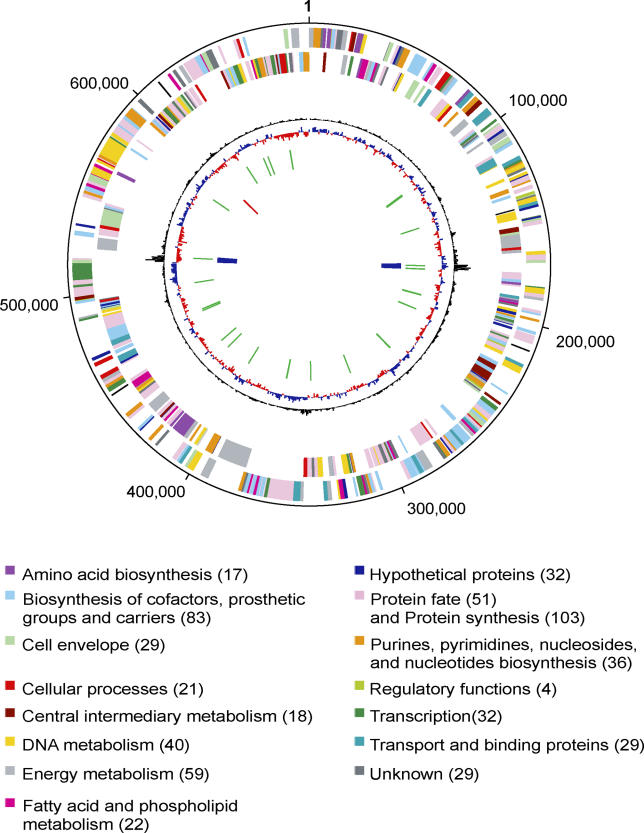
Circular View of the *Baumannia* Genome Circles correspond to the following features, starting with the outermost circle: (1) forward strand genes, (2) reverse strand genes, (3) χ ^2^ deviation of local nucleotide composition from the genome average, (4) GC skew, (5) tRNAs (green lines), (6) rRNAs (blue lines); and (7) small RNAs (red lines). Color legend for CDSs and number of genes in each category are at the bottom.

A total of 46 noncoding RNA genes were identified: six rRNAs (two sets of 16S, 5S, and 23S), one small RNA, and 39 tRNAs including at least one for each of the 20 amino acids. A total of 605 putative protein-coding genes (CDSs) were identified in the genome, and 89.9% of these can be assigned a putative biological function. An overview of the *Baumannia* genome and its encoded genes is illustrated in Figure 1, and features of these genes are summarized in Table S1. Only four of the CDSs lack detectable homologs in GenBank or other complete genomes and thus can be considered “orphan” genes.

### Evolution of *Baumannia* and the Genomes of Intracellular Organisms

**Figure 2:**
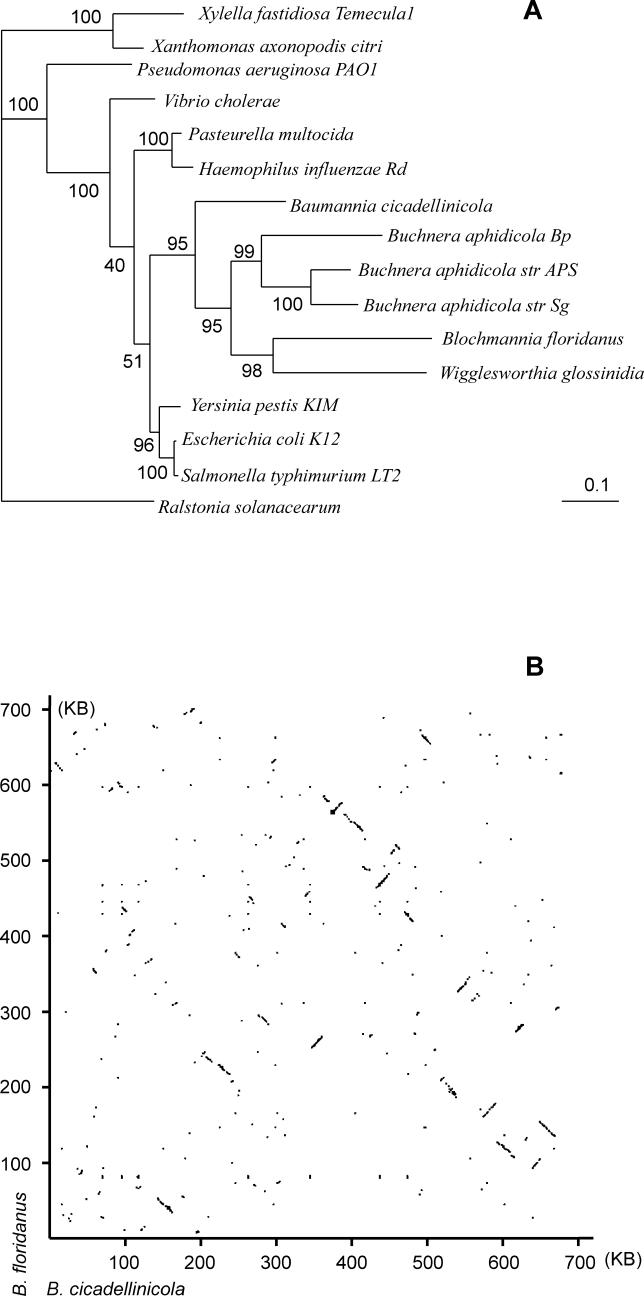
Genome-Based Phylogenetic Analysis of *Baumannia* (A) Maximum-likelihood tree of gamma-proteobacterial endosymbionts. The tree was built from concatenated alignments of 45 ribosomal proteins using the PHYML program. The bootstrap value is based upon 1,000 replications. (B) Gene order comparison of *Baumannia* and Blochmannia floridanus. The plot shows the locations of homologous proteins between the two genomes.

Genome sequences have been found to be very useful in providing for better resolution and accuracy in phylogenetic trees than is achieved using single genes such as rRNA genes [ 17]. Although there are many ways to build genome-based trees, one particularly powerful approach is to identify orthologous genes between species and to combine alignments of these genes into a single alignment. We built a tree for *Baumannia* and related species from 45 ribosomal proteins using this concatenation approach ( Figure 2A). This tree supports the rRNA-based grouping of *Baumannia* with the insect endosymbionts of the genera *Buchnera, Wigglesworthia* (symbionts of tsetse flies) *,* and *Blochmania* (symbionts of ants) [ 16]. However, the branching order is different in the protein tree with *Baumannia* being the deepest branching symbiont. As in prior genomic studies [ 18], the insect endosymbionts in the tree in Figure 2A are monophyletic (i.e., they share a common ancestor to the exclusion of all other species for which genomes are available). A possible close relationship of *Baumannia* to the other symbionts in the group is further supported by the presence of a substantial number of segments of conserved gene order ( Figure 2B).

All of these insect endosymbionts, including *Baumannia,* exhibit many genome-level trends commonly found in intracellular organisms when compared to free-living relatives, including a smaller genome, lower G + C content, a higher average predicted isoelectric point for encoded proteins, and more rapidly evolving proteins ( Table 1, Figure 2A). Of critical importance to understanding these trends is that they occur in all types of intracellular organisms (e.g., mutualists and pathogens) from across the tree of life (archaea, bacteria, and eukaryotes). Much research has focused on trying to understand the mechanisms underlying these trends for which there are two major hypotheses: the loss of DNA repair genes resulting in subsequent changes in mutation patterns or changes in population genetic parameters that lead to more genetic drift [ 19, 20].

As a global explanation, the population genetic forces have more support (e.g., [ 21– 23]), but the issue is far from resolved. One reason for this lack of resolution is that it is usually difficult to reconstruct the early events in the evolution of intracellularity. This insect endosymbiont group has many advantages that have made it a model system for resolving these early events. The addition of the *Baumannia* genome further improves the utility of this group for reasons we detail below.

One limitation of studies of the evolution of intracellular organisms is that the evolutionary separation between free-living and intracellular species is usually very large. For example, although much can be learned about recent mitochondrial evolution by comparative analysis of mitochondrial genomes, it is not even known what subgroup of *Alphaproteobacteria* contains the closest free-living relative of these organelles. This is because the mitochondrial symbiosis originated billions of years ago. The insect endosymbionts lack this limitation both because their symbioses evolved relatively recently and because of the large diversity of genomes available for the *Gammaproteobacteria.* To make the most use of these benefits, it is imperative to have an accurate picture of the phylogeny of the symbionts. The addition of the *Baumannia* genome is useful in this regard because its proteins appear to be evolving more slowly (as indicated by shorter branch lengths in Figure 2A) than those in the other endosymbionts. Having one organism with relatively short branch lengths in this group makes it more likely that the monophyly of the insect endosymbionts in trees is a reflection of their true history and not an artifact of phylogenetic reconstruction known as long-branch attraction.

The branch-length finding is an example of how *Baumannia* can be considered as a “missing link” in that it is an intermediate in many ways between the other insect endosymbionts and free-living species. This is the case not only for branch length but also for phylogenetic position (it is the deepest branching species), isoelectric point (pI), and G + C content ( Table 1). By filling in the gaps between the free-living and intracellular species, the *Baumannia* genome should allow better inferences of the early events in the evolution of intracellularity.

**Figure 3:**
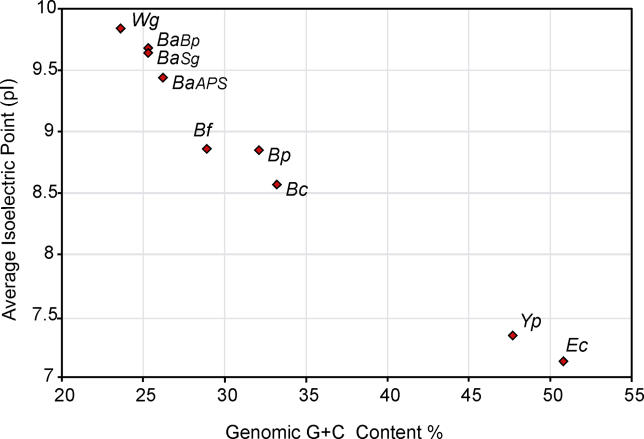
Correlation between Genomic G + C Content and the Average pI of the Proteins of Endosymbiotic and Free-Living *Gammaproteobacteria* Species shown are Buchnera aphidicola APS (Ba _APS_), Buchnera aphidicola BP (Ba _Bp_), Buchnera aphidicola SG (Ba _Sg_), *Baumannia* (Bc), Blochmannia floridanus (Bf), Blochmannia pennsylvanicus (Bp), E. coli K12 (Ec), Wigglesworthia glossindia (Wg), and Yersinia pestis KIM (Yp).

*Baumannia* is not intermediate in value between free-living species and other insect endosymbionts for all “intracellular” features. For example, its genome size is smaller than that of some of the other endosymbionts. This is an important finding since the absolute values for many other features are highly correlated, both in this group and in other symbiont groups [ 24]. An example of this is shown for pI and G + C content ( Figure 3). Another way of looking at this is that the *Baumannia* genome has shrunk more than one might expect based on its other intracellular features. This decoupling of the rates of change of different features can be useful in understanding the patterns of evolution in intracellular species. For example, one explanation for the pattern in *Baumannia* is that although it has experienced more gene loss than some of the other insect endosymbionts, it has maintained the most complete set of DNA repair genes for the group ( Table 2). This retention of repair functions may have slowed its rate of change in other features, such as sequence change. If true, this suggests that, although the general differences between intracellular and free-living species may be due to population genetic forces, the variation among intracellular species may be due in part to variation in DNA repair. Consistent with this is the finding that species with the longest branch lengths in the trees ( *Wigglesworthia* and *Blochmania,* Figure 2A) are those that are missing the mismatch repair genes ( Table 2).

**Table 2:**
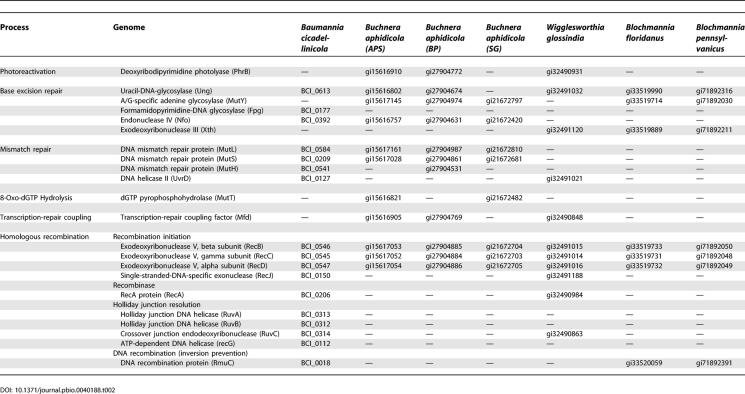
Homologs of Genes Known to Be Involved in DNA Repair and Recombination in the Complete Genomes of Insect Endosymbionts

The differential loss of repair genes among organisms that share many other genome properties allows the insect endosymbiont group to serve as a model for studying the long-term effects of loss of various repair processes. For example, the consequences for genome evolution of losing *recA* can be examined by comparing *Baumannia* and *Wigglesworthia,* which retain it, to *Buchnera,* which lack it. The same logic can be used to study the effects of the loss of the DNA replication initiation gene *dnaA* which is missing from *Baumannia* (see above), *Wigglesworthia,* and *Blochmannia* [ 18, 25] but is present in the other insect endosymbionts. Although the species without *recA* may be able to survive with little or no recombination, those lacking *dnaA* must make use of alternative initiation pathways. Some alternatives such as pathways based on *priA* and *recA* [ 26] can be ruled out since at least one of these is missing from each of the species missing *dnaA*. The *recBCD* genes may play some role in initiation. This would explain why the *recBCD* genes are present in all insect endosymbionts ( Table 2) including those missing *recA*, which is required for the “normal” role of *recBCD* in recombination.

### Single Nucleotide Polymorphisms Are Abundant in the *Baumannia* Population

Genetic variation among individuals is both a complication of genome sequencing projects of uncultured species and a valuable source of information about microbial populations. For the *Baumannia* data, we used a stringent search protocol that may have missed some true polymorphisms but should have eliminated variation that was due to sequencing errors or cloning artifacts (see Materials and Methods). In total, we identified 104 single nucleotide polymorphisms (SNPs) and two insertion-deletion differences (indels) that fit these criteria. Details of the locations and types of polymorphisms are given in Table 3.

**Table 3:**
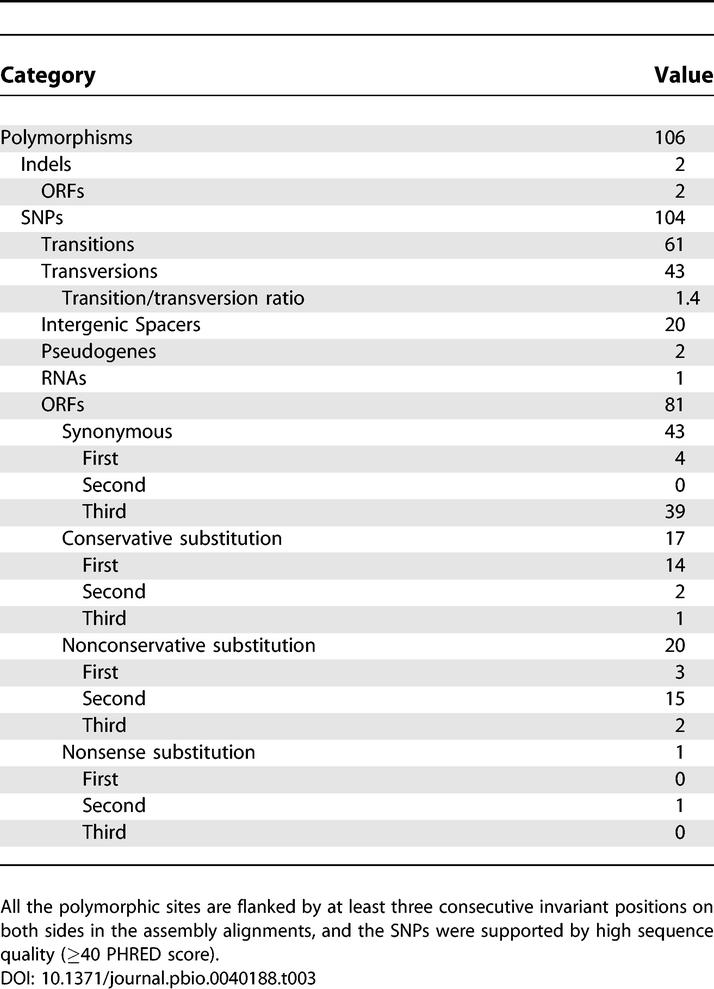
Categorizations of Polymorphisms Detected in the Assembled *Baumannia* Genome

Since our DNA was isolated from the symbionts of hundreds of hosts, one major question is whether the observed polymorphisms were between symbionts within one host or between hosts. We used polymerase chain reaction surveys of individual insects to address this question. Of the 40 insects for which sequences were obtained individually for two loci, 35 showed identity to the consensus sequence for the *Baumannia* genome and five possessed the alternative alleles that were present as minority bases at four sites (two per fragment). No polymorphism was detected within individual hosts. Thus, the polymorphisms that we identified are real, and they reflect differences between symbionts of different hosts.

Since the *Baumannia* can be treated as maternally inherited markers, the finding of significant levels of polymorphism between hosts suggests that the sampled population contains individuals from two separate origins. This is somewhat in conflict with theories suggesting a single introduction of a small number of individuals into California [ 10] but is consistent with results from recent mitochondrial analysis [ 27].

Sequence polymorphisms have been detected in genomic studies of other insect endosymbionts [ 3, 28]. The most relevant one for comparison to *Baumannia* is a study of the ant endosymbiont *Blochmannia pennsylvanicus,* although we note that the criteria they used for detecting a polymorphism were somewhat less stringent than ours [ 28]. The percentage of the SNPs that are in coding regions is different in the two species (81% in *Baumannia* and 65% in B. pennsylvanicus), but this is in line with differences in gene-coding density (88% in *Baumannia* and 76% in B. pennsylvanicus). For both species, the percentage of SNPs in protein-coding genes that represent synonymous differences is higher than expected from random changes given the genomic base compositions (52% in *Baumannia* and 62% in B. pennsylvanicus). This indicates ongoing purifying selection in both genomes. The most significant difference between the species is the higher ratio of transitions to transversions in B. pennsylvanicus (2.9 versus 1.4 in *Baumannia;* Table 3). We propose that this is due to the absence of mismatch repair genes in B. pennsylvanicus (as discussed above) *,* which in other species leads to an increase in transition mutations [ 29]. An absence of mismatch repair would also explain the higher incidence of indels in B. pennsylvanicus.

### Metabolic Reconstructions Provide Insight into the Biology of *Baumannia*

**Figure 4:**
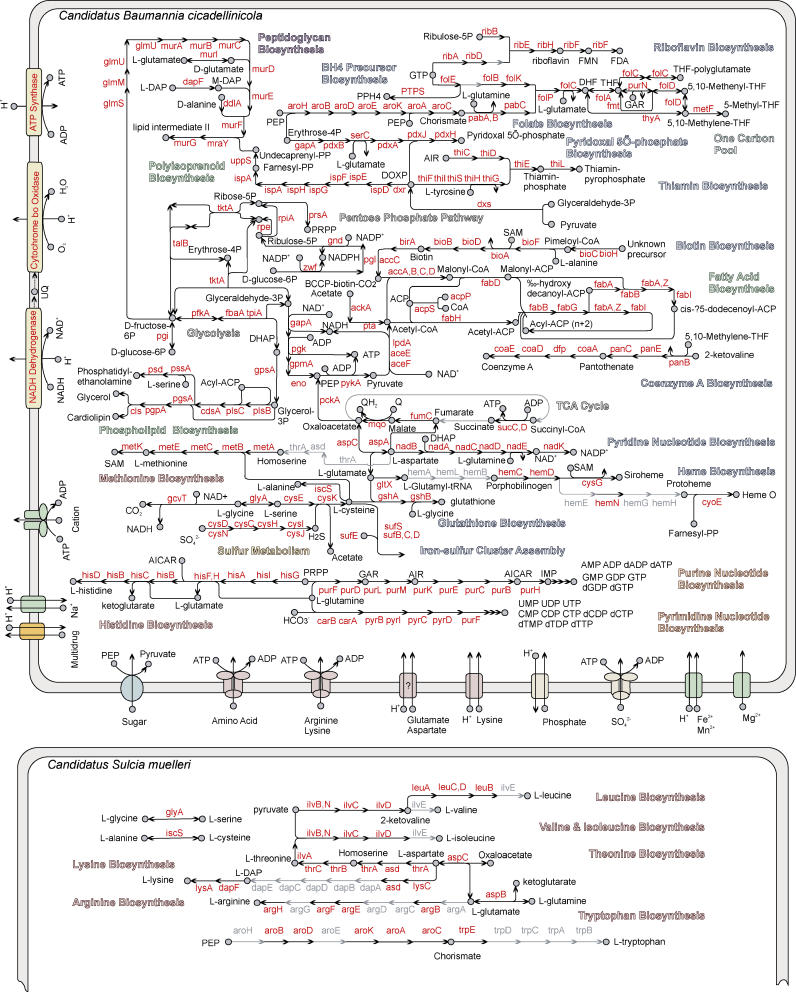
Predicted Metabolic Pathways in *Baumannia* and the Predicted Amino Acid Biosynthesis Pathways Encoded by the Partial Genome Sequence of *Sulcia* Genes that are present are in red and the corresponding catalytic pathways are illustrated in solid black lines; the genes that are absent in the *Baumannia* genome and genes that have not been identified in the partial *Sulcia* genome are in gray, and the corresponding metabolic steps are illustrated in gray lines.

Predictions of the metabolism of an organism from its genome sequence are critical to studies of uncultured organisms because of the difficulty of experimental studies. We have generated such a prediction for *Baumannia* ( Figure 4). Although all such predictions should be viewed as hypotheses, not facts, they are greatly improved by having closely related species for which experimental studies are available. This is yet another advantage of working on the insect symbionts in the *Gammaproteobacteria*. For example, almost all *Baumannia* genes have clearcut orthologs in well-studied organisms such as Escherichia coli.

As expected, based on its small genome size, *Baumannia* has a relatively limited repertoire of synthetic capabilities. There are some important features of its predicted metabolism, and we discuss these in this and the next few sections of this paper, calling attention in particular to those of relevance to the host-symbiont interaction.

*Baumannia* is predicted to synthesize its own cell wall and plasma membrane, processes known to be lost in some intracellular species. It is, however, apparently unable to synthesize the lipopolysaccharide (LPS) commonly found in the outer membrane of other Gram-negative bacteria. The same is true for *Buchnera* species but not for *Wigglesworthia* and *Blochmannia*. The functional significance of this difference is unclear. On one hand, lipid A (the lipid component of the LPS) is generally highly toxic to animal cells; thus, LPS may be disadvantageous for endosymbionts and discarded during their evolution. Alternatively, the difference may reflect differences in the packaging of symbionts within the host bacteriocytes. *Buchnera* and *Baumannia* cells are surrounded by host-derived vesicles, while *Wigglesworthia* and *Blochmannia* directly contact the host cytoplasm.

The findings in regard to sugar metabolism are consistent with *Baumannia* acquiring sugars from its host and using them for energy metabolism. For import, a complete mannose phosphotransferase permease system is present including an Enzyme II ^Man^ complex, the phosphotransferase system Enzyme I, and histidyl phosphorylatable protein PtsH. Imported sugars could then be fed into glycolysis. However, since the tricarboxylic acid cycle appears to be incomplete, apparently reducing power must come from other sources such as glycolysis itself, a pyruvate dehydrogenase complex, and an *mqo* type malate dehydrogenase. An intact electron transport chain consisting of NADH dehydrogenase I, cytochrome *o* oxidase, and ATP synthase is present.

The most striking aspects of the metabolism of *Baumannia* relates to what it apparently does and does not do in terms of the synthesis of essential nutrients missing from the hosts' xylem diet.

### *Baumannia* Is a Vitamin and Cofactor Machine

A large fraction of the *Baumannia* genome (83 genes, 13.7% of the total) encodes proteins predicted to have roles in pathways for the synthesis of a diverse set of vitamins, cofactors, prosthetic groups and related compounds ( Figure 4, Table S1). These include thiamine (vitamin B _1_), riboflavin (vitamin B _2_), niacin (vitamin B _3_), pantothenic acid (vitamin B _5_), pyridoxine (vitamin B _6_), as well as biotin and folic acid. More detail on the pathways and the basis for the predictions is given below.

For the synthesis of riboflavin, folate, pyridoxal 5′-phosphate, and thiamine, complete pathways for de novo synthesis could be identified with *Baumannia*'s ability to produce endogenously important precursors such as ribulose-5-phosphate, phosphoenolpyruvate, pyruvate, dihydroxyacetonephosphate, glyceraldehyde-3-phosphate, erythrose-4-phosphate, guanosine triphosphate, 5-aminoimidazole ribonucleotide, 5′-phosphoribosylglycinamide, and 5,10-methylene-tetrahydrofolate.

For some compounds, although homologs of enzymes carrying out key steps in other species could not be identified, candidates for alternatives are present suggesting the pathways are complete. For example, the step normally carried out by erythrose 4-phosphate dehydrogenase *(Epd)* in the pyridoxal 5′-phosphate pathway might be carried out by glyceraldehyde 3-phosphate dehydrogenase (GapA) as seen in some other species [ 30].

There are some compounds for which we could identify homologs of all known genes in biosynthetic pathways. However, some enzymes in these pathways are still unknown in any organism, and thus we could not identify them here. This is true for the pyrimidine phosphatase in the riboflavin pathway and the dihydroneopterin monophosphate dephosphorylase in the folic acid pathway. We believe it is likely that these pathways are complete in *Baumannia* and that, due to its ultracompact gene pool, *Baumannia* provides an ideal opportunity to identify the genes encoding the enzymes for these steps.

Perhaps most interesting are the pathways for which we could identify genes underlying many downstream steps but for which *Baumannia* would need to import some intermediates to feed those steps. For example, *Baumannia* encodes genes for the last three steps for siroheme synthesis, and the last step of heme O pathway, but candidate genes underlying the upstream steps could not be identified. Thus, *Baumannia* needs to import prophobilinogen and protoheme as substrates for these incomplete pathways. This pattern is particularly apparent in that *Baumannia* appears to be able to synthesize many cofactors from amino acids but is unable to synthesize the amino acid precursors. Examples of such pathways and the amino acid required include thiamin (tyrosine), biotin (alanine), pyridine nucleotides (aspartate), and folate and pyridoxal 5′-phosphate (glutamine and glutamate). This suggests that *Baumannia* must import these amino acids. The lack of amino acid biosynthesis pathways also makes it a necessity for *Baumannia* to import 2-ketovaline as a precursor for the synthesis of pantothenate and coenzyme A.

Due to the diversity of vitamin and cofactor synthesis pathways that are present, we conclude that *Baumannia* is providing its host with these compounds due to their low abundance in its diet. In this respect *Baumannia* is more similar to *Wigglesworthia,* the symbiont of tsetse flies, than to *Buchnera*.

### Amino Acid Biosynthetic Pathways Are Generally Absent from *Baumannia* and Likely Are Found in Another Organism in the System

In contrast to what is seen for vitamin and cofactor synthesis, *Baumannia* is predicted to encode a very limited set of amino acid synthesis pathways. The few capabilities that are present include histidine biosynthesis, synthesis of methionine if external homoserine is provided, and the ability to make chorismate but not to use it as substrate for production of aromatic amino acids as in most bacterial species. Except for histidine, no complete pathways for the synthesis of any amino acids essential to the host are present.

The lack of amino acid synthesis pathways is apparently compensated by an ability to import amino acids from the environment using a general amino acid ABC transporter, an arginine/lysine ABC transporter, a lysine permease, and a proton/sodium-glutamate symport protein, although the gene for the latter is disrupted by one frameshift. The import of amino acids is apparently used not just for making proteins but also for energy metabolism. The latter is evident by the presence of the aspartate ammonia-lyase AspA, which could be used to convert l-aspartate to fumarate, which in turn can be fed into the tricarboxylic acid cycle.

The absence of essential amino acid synthesis pathways from *Baumannia* implies that both the host and *Baumannia* must obtain amino acids from some external source or sources. The sole diet of H. coagulata is xylem sap [ 10], in which essential amino acids are rare to absent; however, a substantial portion of the nitrogen in xylem occurs in the form of certain nonessential amino acids, including glutamine, aspartic acid, and asparagine (e.g., [ 11, 14, 31, 32]). The essential amino acid synthesis pathways have not been found in any animal species studied to date, and nutritional studies in insects indicate that these compounds are required nutrients in insects as in mammals. Thus, the most plausible alternative is that another organism that is reliably present in the “ecosystem” of the host body is synthesizing the missing amino acids.

### Analysis of Leftover Shotgun Sequence Reads Reveals the Presence of Amino Acid Synthesis Genes in Organisms Other than *Baumannia*

The most likely candidate for another organismal source of the amino acid synthesis pathways is *Sulcia,* the other coevolving symbiont found in bacteriomes mentioned above. Although we did not set out to sequence the *Sulcia* genome as part of this project, we realized we might have inadvertently acquired some of it since many sequence reads from the shotgun sequencing did not assemble with the *Baumannia* genome. These reads derived from cells of other organisms that were present in the tissue samples we used to isolate DNA for the *Baumannia* sequencing. An initial search of these sequence reads revealed the presence of homologs of genes with roles in the synthesis of essential amino acids. However, we could not conclude that these reads were from *Sulcia,* since there could have been cells of other organisms in the sample as well. To sort the extra reads into taxonomic bins, we adapted methods we have used to sort sequences from environmental shotgun sequencing projects (see Materials and Methods) and were able to assign non- *Baumannia* sequences to three main groups: host, *Wolbachia* related, and *Sulcia* related.

The finding of some *Wolbachia* in the sample was not surprising since rRNA surveys have shown that these alphaproteobacterial relatives of Rickettsia are found in many sharpshooters including *H. coagulata.* We note that we did not detect any sequences from the previously sequenced phytopathogen *X. fastidiosa,* which colonizes the surface of the foregut and is not present in the bacteriomes that we used for DNA isolation. In addition, although some of our sequences show high identity to sequences annotated as being from a phytoplasma, we believe this annotation is incorrect. The “phytoplasma” DNA was isolated from the saliva of the leafhopper Orosius albicinctus [ 33]. However, all the sequences in our sample that showed matches to sequences annotated as “phytoplasma”-like show phylogenetic relationships to the *Bacteroidetes* phylum. In addition, *Sulcia* is known to be a symbiont of species in the Deltocephalinae, the leafhopper subfamily containing O. albicinctus [ 9]. Thus, the putative “phytoplasma”-like sequences with matches in our sample are likely from the *Sulcia* symbiont of O. albicinctus. Why these sequences appeared in samples from salivary secretions is unclear.

### Amino Acid Synthesis Pathways Are in *Sulcia* and Not Other Organisms in the Sample

**Figure 5:**
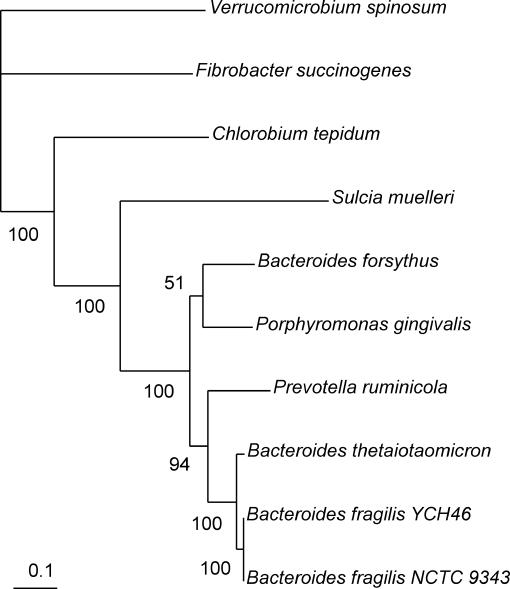
Maximum-Likelihood Tree of *Sulcia* with Species in the *Bacteroides and Chlorobi* Phyla for which Complete Genomes Are Available The tree was build using the PHYML program from the concatenated alignments of 34 ribosomal proteins. The bootstrap values are based upon 1,000 replications.

Of the essential amino acid synthesis genes identified in the extra shotgun sequence reads, the vast majority (31 of 32) were assigned to the *Sulcia* bin. In contrast, only one gene ( *argB*) was found in the *Wolbachia* bin and none were found in the host bin. We therefore sought to obtain as much sequence information as possible from the *Sulcia* symbionts in this system. First, we completed the sequence of all plasmid clones for which at least one read had been assigned to the *Sulcia* bin. In addition, we constructed a new library from tissue thought to contain more of the *Sulcia* symbiont than the library used for the initial sequencing. End-sequencing of this library identified some additional *Sulcia-*derived clones, and these, too, were completely sequenced. After conducting another round of assembly and assigning contigs and sequences to taxonomic bins, we were able to assign 146,384 bp of unique sequence to *Sulcia*. In these data, we identified 166 protein-coding genes. A phylogenetic analysis of a concatenated alignment of ribosomal proteins groups this protein set within the Bacteroidetes, thus supporting our assignment of these sequences to *Sulcia* ( Figure 5).

Although theoretically we could obtain a complete genome sequence of *Sulcia* by very deep sequencing of the samples we have obtained, this was not practical given limited funds. Nevertheless, analysis of the incomplete genome is quite revealing. First, among the 166 predicted proteins are 31 that underlie steps or whole pathways for the synthesis of amino acids essential for the host ( Figure 4). These include the complete pathway of threonine biosynthesis and nearly complete pathways for the synthesis of leucine, valine, and isoleucine (the only gene not sampled is *ilvE* encoding the branched chain amino acid aminotransferase). In addition, multiple genes in the pathways for the synthesis of lysine, arginine, and tryptophan are present. We believe it is likely that these pathways are present and that the missing genes are in the unsequenced parts of the genome.

One question that remains is where *Sulcia* gets all of the nitrogen for these amino acids. One possibility is that it acquires and then converts nitrogenous organic compounds, particularly the nonessential amino acids known to be present in xylem (e.g., [ 14, 32]). Alternatively, it is possible that *Sulcia* assimilates nitrogen from compounds such as ureides or ammonium, which are found in xylem (e.g., [ 14, 32, 34]). It has been proposed that *X. fastidiosa,* the plant pathogen vectored by *H. coagulata,* makes use of the ammonium in xylem as a nitrogen source [ 35]. Alternatively, *Sulcia* could garner inorganic nitrogen from the host, for which ammonium is a waste product [ 10, 13]. Host waste is apparently is a source of nitrogen for *Blattabacterium,* close relatives of *Sulcia* that are symbionts of cockroaches [ 36]. Although some insect genomes encode enzymes that may allow for this (e.g., glutamine synthetase or glutamate synthase (e.g., [ 37]), it is not yet known whether these capabilities are present in sharpshooters. Whatever the source of its nitrogen, the genome analysis indicates that *Sulcia* apparently can make the amino acids required by the host.

The other abundant organism in our DNA was *Wolbachia,* an unlikely candidate as the source of these compounds. *Wolbachia* cannot be an obligate symbiont of sharpshooters because it infects only some individuals. Screening of individual H. coagulata indicates that some do not contain *Wolbachia* ([ 16], two of 40 insects were uninfected in our screens); and screening of individuals of the closely related species, Homalodisca literata (a synonym of H. lacerta), revealed no cases of *Wolbachia* infection. Also, although we have sampled only a fraction of the *Wolbachia* genome, the absence of amino acid synthesis pathways is consistent with the complete lack of essential amino acid biosynthesis in any of several sequenced *Wolbachia* genomes (two complete and many incomplete) [ 23, 38, 39].

We therefore conclude that *Sulcia* is most likely the sole provider of essential amino acids for *H. coagulata.* Thus, this member of the *Bacteroidetes* phylum appears to function in a similar way to *Buchnera* and *Blochmannia* species in the *Gammaproteobacteria*.

### *Sulcia* and *Baumannia* Complement Each Other

**Figure 6:**
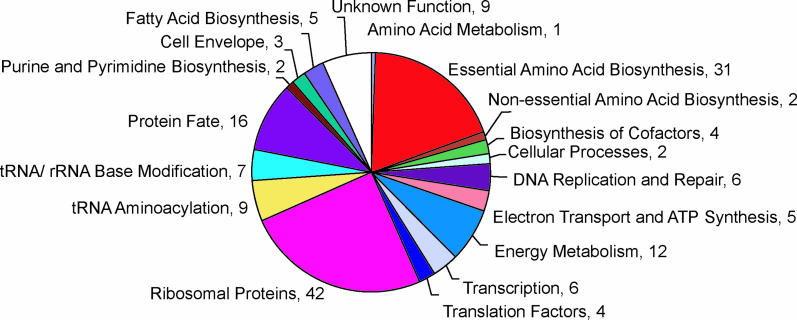
The Distribution into Functional Role Categories of the 166 Predicted Genes Encoded in the 146,384-bp Partial Sequence of the *Sulcia* Genome Data are shown for all ORFs that encode proteins longer than 45 amino acids that have BLASTP matches with an E-value less than 10 ^−3^ to proteins in complete genomes. Different fragments of the same gene are counted as one gene in the chart.

We found very few genes in the partial *Sulcia* genome for vitamin or cofactor synthesis. Since the *Sulcia* genome appears to be quite small and we have apparently sampled a large fraction of it, we can speculate that few such genes are likely to be present. Thus, in the 146 kb of sequence assigned to *Sulcia,* we have already found many of the core housekeeping types of genes (e.g., 40 ribosomal proteins and ten tRNA synthetases ( Figure 6, Table S2). A very small genome size is consistent with phylogenetic reconstructions indicating that *Sulcia* is an extremely old symbiont, originating in the Permian [ 9].

The paucity of vitamin and cofactor synthesis pathways in *Sulcia* suggests the possibility that *Sulcia* and *Baumannia* play complementary, nonoverlapping roles in this symbiotic system. Not only do they appear to provide different resources for the host ( *Sulcia* provides the amino acids and *Baumannia* the vitamins and cofactors) but, based on the current evidence, each does not provide the resources made by the other ( Table 4). Indeed, the single essential amino acid biosynthetic pathway present in the *Baumannia* genome, that for histidine, is correspondingly the sole essential amino acid pathway with multiple steps for which no genes were detected in *Sulcia*. Thus, although *Baumannia* and the host apparently depend on *Sulcia* for the majority of essential amino acids, *Sulcia* and the host may depend on *Baumannia* for histidine. The complementarity between host and each symbiont extends to mutual dependence between the symbionts, which appear to depend on each other for these required compounds and for intermediates in other metabolic processes. For example, we predict that *Sulcia* can make homoserine, which, as discussed above, could be the substrate for methionine synthesis in *Baumannia*. In addition, the valine pathway in *Sulcia* could be the source of the 2-ketovaline for pantothenate and coenzyme A biosynthesis in *Baumannia*. Exchange of intermediates may be occurring for many aspects of metabolism. In the case of ubiquinone, a key component of the electron transport chain, *Baumannia* lacks genes encoding the needed biosynthetic enzymes and thus likely needs to import ubiquinone. The same appears to be true for menaquinone. Strikingly, even though only four of the 166 proteins in *Sulcia* are predicted to be involved in pathways of cofactor synthesis, two are for production of menaquinone and ubiquinone production, which are among the few cofactors whose synthesis is not carried out by *Baumannia*.

**Table 4:**
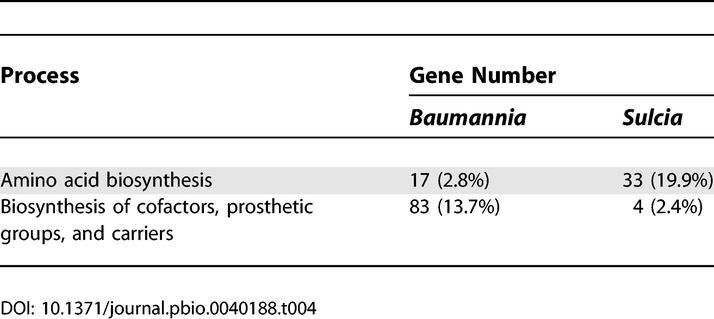
The Complementarity of Amino Acid Biosynthesis and Cofactor Biosynthesis Pathways between *Baumannia* and *Sulcia*

**Figure 7:**
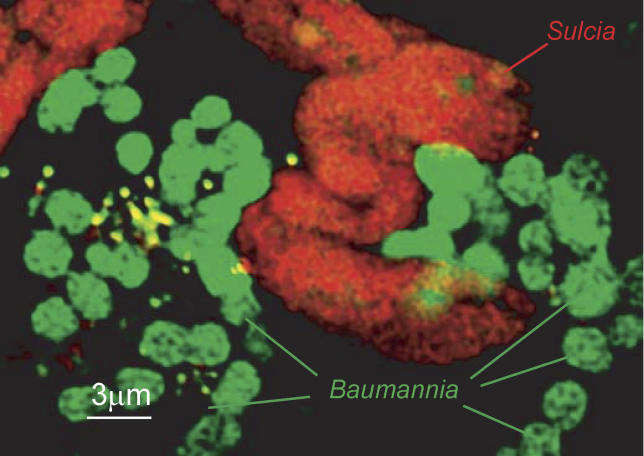
Baumannia and *Sulcia* Coinhabit the Bacteriomes of the Host Insects Fluorescent in situ hybridizations were performed using oligonucleotide probes designed to hybridize selectively to the ribosomal RNA of *Baumannia* (green) and of *Sulcia* (red), respectively. Bacteriomes were obtained from Homalodisca literata (a very close relative of H. coagulata).

The coresidence of *Sulcia* and *Baumannia*, presented here from *H. coagulata,* is representative of a symbiotic pair that is distributed in most or all sharpshooters, a xylem-feeding insect group [ 9, 16]. Thus, the possibility of metabolic complementarity that is suggested by the genome analyses reflects long coevolution of the three lineages represented by the insects and the two bacteria. The two symbionts occur in close proximity within the yellow portion of the host bacteriomes [ 16], and *Baumannia* cells often appear to adhere to the surface of the much larger *Sulcia* cells. This arrangement is illustrated in images from our in situ hybridizations for H. literata, a close relative of H. coagulata ( Figure 7).

### Conclusions

The glassy-winged sharpshooter, *H. coagulata,* feeds on xylem sap, which has very low levels of many nutrients required by insects and other animals [ 10]. Sequence analysis suggests the occurrence of an obligate symbiosis among three organisms: H. coagulata, the gamma-proteobacterial endosymbiont *Baumannia,* and the *Bacteroidetes* bacterial symbiont *Sulcia*. The two bacterial symbionts co-occur within the cytosol of sharpshooter bacteriocytes, sometimes residing within the same cells. The main function of *Baumannia,* as revealed by its genomic sequence, is to provide cofactors, especially water-soluble B-family vitamins, to the host. Partial sequences from *Sulcia* suggest that it provides essential amino acids to the host. The two endosymbionts appear to show functional complementarity and show little overlap in biosynthetic pathways, although full sequencing of the *Sulcia* genome is needed for a comprehensive view of the contributions of these two organisms. Our analysis shows the added insight possible from assigning sequences to organisms rather than treating environmental samples as a representative of a communal gene set.

Many questions remain regarding this fusion of separate lineages into a single metabolic system. For example, the different organisms must balance their contributions to the shared metabolism through coordinated growth and gene expression, and the mechanisms underlying this integration are not known. Also, these bacterial genomes have undergone major reduction in size while apparently maintaining their complementary capabilities, raising the question of how the steps in genome reduction have been coordinated. The sharpshooters and their obligate bacterial endosymbionts provide a simple model of genomic coevolution, a process that has likely been central in the evolution of most organisms living in stable associations.

## Materials and Methods

### Isolation of DNA for sequencing.

The material for sequencing was obtained from adults of H. coagulata collected in a lemon orchard in Riverside, California, in June 2001 and June 2004. The California population was introduced from southeastern United States, Texas, or Mexico within the past 20 years [ 10, 27]. DNA was isolated by first dissecting out the red portion of the bacteriome, which contains mainly *Baumannia* [ 16]. Approximately 200 adults were dissected, in PA buffer, and kept on ice. Immediately following the dissection, the bacteriome samples were disrupted with a pestle and were passaged in PA buffer through a 20-μm filter and then through an 11-μm filter, on ice. The filtering was intended to remove nuclei of the host insect cells. DNA was isolated from the filtered material using standard methods [ 16]. For the second sample, adults were collected in 2004 from the same lemon orchard as before; in this case, we attempted to increase representation of the *Sulcia* genome by dissecting out the yellow portion of the bacteriome from approximately 200 adults and then processing as for the first sample.

### Library construction and shotgun sequencing.

DNA libraries were constructed by shearing the genomic DNA through nebulization, cutting DNA of a particular size out of an agarose gel, and cloning it into the pHOS2 plasmid vector. Then 13,926 sequencing reads were generated from a 3- to 4-kb insert-sized library that was constructed using the first “red bacteriome” DNA sample. In addition, a large insert library (10- to 12-kb inserts) was constructed with DNA purified from the second “yellow bacteriome” DNA sample and 3,396 reads were generated from this library. In order to get more sequences to close the *Baumannia* genome and finish *Sulcia* clones, 2,986 sequencing reads were generated in the closure efforts.

### Assembly and closure of the *Baumannia* genome.

The shotgun sequence data were assembled using the TIGR assembler [ 40], and the genome of *Baumannia* was closed using a combination of primer walking, multiplex PCR, and generation and sequencing of transposon-tagged libraries. Repeats were identified using RepeatFinder [ 41], and sequence and assembly of the repeats were confirmed using PCRs that spanned the repeat. The final assembly was checked such that every single base is covered by at least two clones and has been sequenced at least once in each direction. The average depth of coverage for the genome is 6.4. A putative origin of replication was identified by analysis of transitions in oligonucleotide skew [ 42].

### Identification and sequence of fragments of the genome of *Sulcia*

Sequence reads from the shotgun sequencing data that did not map to the *Baumannia* genome were processed to sort them into candidate taxonomic groups (bins). First, they were assembled into contigs (although the vast majority of sequences did not assemble). Then each contig was analyzed to assign it to a putative bin using a combination of BLAST searches and phylogenetic trees. All sequences were searched with BLASTN and BLASTX against multiple sequence databases to identify top scoring matches. In addition, the BLASTX search results were used to identify possible proteins encoded in the sequences; these proteins were then used to build phylogenetic trees. The taxonomic identity of the nearest neighbor in these trees was extracted and stored. From these search results, sequences were assigned to taxonomic groupings of as low a taxonomic level as possible (e.g., if a protein grouped within a clade of sequences from insects, it was assigned to an insect bin). Examination of the results revealed that there were three major bins: insect, *Wolbachia,* and *Bacteroidetes.* There were also many sequences that were not readily assignable to one of these bins but could be assigned to higher level groups such as “Bacteria.” Based on rRNA studies and other work, we assumed that all sequences that were assigned to animals were likely from the host, and that all assigned to *Bacteroidetes* were likely from the *Sulcia* symbiont. Thus we refer to these bins as host and *Sulcia,* respectively.

Initial analysis indicated that there were some genes encoding proteins predicted to be involved in amino acid synthesis in the *Sulcia* bin. In order to get more data from this taxonomic group, we decided to finish sequencing any clones that mapped to this group and that were at the end of contigs. In order to reduce the probability of wasting funds sequencing clones from another organism, we developed more stringent criteria for selecting which of the initial *Sulcia* bin sequences to characterize further. In these criteria, at least one of the following had to be true: (1) part of the contig contained a match of greater than 99% identity to the previously sequenced 16S to 23S rDNA of *Sulcia* [ 16]; (2) BLASTP searches of translated sequences against all complete microbial genomes gave a best match (based on E-value) to a member of the *Bacteroidetes* phylum; (3) predicted proteins branched with genes from *Bacteroidetes* species in neighbor joining trees; and (4) the sequences were significantly AT biased. For all sequence reads that were assigned to *Sulcia* using these criteria, if they were at the end of a contig, the remainder of the clone was sequenced.

After this additional sequencing, all sequence reads (including the new reads) that did not map to the *Baumannia* genome were reassembled using the Celera Assembler. From this new assembly, a “final” list of contigs likely to be from *Sulcia* was identified using similar criteria as above: first, the fragment had to have ORFs that either had a best scoring BLAST hit to a sequence in the *Bacteroidetes* phylum or position next to a *Bacteroidetes* gene in neighbor-joining trees of the proteins identified by BLASTP. In addition, GC content had to either be below 40% or the fragment had to have greater than 99% match for at least 200 bases to the previously sequenced 16S rDNA of the *Bacteroidetes* endosymbiont of the H. coagulata. The low GC content criterion was applied to exclude contamination from free-living bacteria in our DNA sample.

From the new assemblies, contigs we also reassigned to the *Wolbachia* and host bins. To be considered to be from *Wolbachia,* the contig had to have not been assigned to *Baumannia* or *Sulcia* and had to have a top BLASTX hit to sequences from other *Wolbachia*. In total, 43,079 bp of unique sequence were assigned to *Wolbachia*. Another 120 kb worth of sequences and assemblies could not be assigned conclusively to *Sulcia*, *Wolbachia,* or *Baumannia* but had top BLAST hits to bacterial genes.

### Genome annotation.

For the *Baumannia* genome, the GLIMMER program was used to identify putative CDSs [ 43]. Some putative CDSs were discarded if they had no significant sequence similarity to known genes and if they had significant overlaps with other CDSs with significant sequence similarity to known genes. Noncoding RNAs were identified as described previously [ 23]. Gene function annotation was based on results of BLASTP searches against Genpept and completed microbial genome and hidden Markov model searches of the PFAM and TIGRFAM databases [ 44, 45]. We identified only four genes in the *Baumannia* genome that did not have BLASTP matches to any protein entries in Genpept or proteins from publicly available complete genomic sequences (using an E-value cutoff of 0.01). GC skew and nucleotide composition analysis were performed as described previously [ 23].

For the partial *Sulcia* genome, ORFs were identified using the EMBOSS package [ 46]. Only those predicted peptides that were larger than 45 amino acids in length and that had BLASTP hits against microbial genome databases at E-value cutoff of 0.001 were kept as potential genes. The functional annotation of the *Sulcia* genes is mostly based on the top BLASTP hits.

### DNA polymorphisms in *Baumannia*

Polymorphism analysis was done on the results of the initial assemblies of the shotgun sequence data. Finished sequences were not used since these were based on part on targeted sequencing of select clones, which eliminates the random nature found in the shotgun data. SNPs and indels were identified using stringent criteria to identify regions with variation among sequence reads that were not likely due to sequencing errors.

A site was considered to have an SNP if (1) it had high sequence quality (≥40 PHRED score); (2) the assembly column in which it was found had more than 4-fold coverage; (3) it had differences among the reads at that position, and (4) the variable site was adjacent to at least three invariant positions on both sides. We used only positions that did not have variable flanking sites to prevent alignment errors from mistakenly causing us to score a site as polymorphic. SNPs in coding regions were characterized as synonymous (no amino acid change), conservative (common amino acid change), nonconservative (unusual amino acid chance), or nonsense (stop codon), with a BLOSUM80 matrix being used to distinguish conservative from nonconservative.

Alignment gaps were scored as INDELs only if (1) the column with the gap had at least 4-fold coverage; (2) the aligned column had at least two high-quality sequence reads (≥40 PHRED score), and (3) three consecutive sequence reads on both sides of the gap(s) were of high quality (≥40 PHRED score).

To determine whether the polymorphisms occurred within or between individual host insects, DNA was extracted from the bacteriomes of 40 individual *H. coagulata*. These individuals were from the same collection that was used for the genomic sequences and had been frozen at −80 °C at the time of collection. PCR primers were designed for two regions (554 bp and 725 bp) that contained SNPs. These regions were amplified, the reaction products cleaned with Qiagen (Valencia, California, United States) miniprep columns, and the products were sequenced directly in both directions at the University of Arizona Genomic Analysis and Technology Center using an ABI 3730 sequencing machine.

We also used these 40 individuals to determine whether *Wolbachia*, which was detected in our sequence dataset, was present in all insects in the population. This determination was made on the basis of diagnostic PCR based on two genes, 16S rRNA and *wsp*, with the *Baumannia* SNP loci described above used as controls for DNA quality. Individuals with products for both *Wolbachia* loci were scored as positive, and individuals lacking both were scored as negative. (No individuals yielded one product and not the other.)

### Comparative genomics.

The predicted proteomes of *Baumannia, Wigglesworthia, Blochmannia,* and three strains of *Buchnera* were combined into one database. “All vs. all” BLASTP searches were performed for this database, and a Lek clustering algorithm was applied to cluster the peptides into gene families. An E-value cutoff of 1 × 10 ^−4^ for the BLASTP results and a Lek similarity cutoff of 0.6 were chosen for the gene family clustering [ 47]. All the genes were searched against PFAM and the TIGRFAM database by HMMER, as well as against the reference genomes of E. coli K12 and Yersinia pestis KIM by BLASTP. Gene families were curated and functional roles were assigned according to the HMM and BLASTP search results.

Whole genome alignments of *Baumannia* versus *Wigglesworthia*, *Blochmannia* **,** three strains of *Buchnera, E. coli,* and *Yersinia pestis* were performed. Genome alignments were built using the BLASTP-based Java program DAGCHAINER [ 48] with an E-value cutoff of 1 × 10 ^−5.^

### Phylogenetic analysis.

A set of 45 ribosomal protein genes for which orthologs could be identified in *Baumannia* and other genomes of interest was selected. Each ortholog set was aligned using CLUSTALW, the alignments were concatenated, a maximum likelihood tree was built by PHYML, and 1,000 bootstrap replicates were performed [ 49]. The same approach was adapted for building the maximum likelihood tree from a set of 34 ribosomal protein genes for *Sulcia* and selected genomes of interest.

### Pathway analysis.

The proteomes from *Baumannia* and *Sulcia* were searched against KEGG GENES/SSDB/KO [ 50] databases by BLASTP. Neighbor-joining trees were built by QUICKTREE [ 51], and EC numbers were assigned to the *Baumannia* proteins basing on the nearest neighbor in the phylogenetic trees. The list of the EC number present in the *Baumannia* genome was submitted to the KEGG Web site ( http://www.genome.jp/kegg) to obtain all the potential pathways in the genome. Each pathway was examined and verified according to our genome annotations as well as the pathway descriptions in the EcoCyc database [ 52].

### Fluorescent in situ hybridizations to visualize coresiding symbionts.

In order to obtain images of the symbionts and to verify the correspondence of 16S rDNA sequences to the organisms inhabiting bacteriomes, these structures were dissected from newly collected *H. literata,* a close relative of H. coagulata that occurs in Tucson, Arizona. (This procedure requires live material, and H. coagulata is a major pest that is not yet established in Arizona where this work was carried out.) Bacteriomes were disrupted in buffer, hybridized, and visualized as described in [ 9], except that mounts were made in antifading Vectashield medium (Vector Laboratories, Burlingame, California, United States), and the microscope and software used were Deltavision RT and SofWoRx V2.50 Suite V1.0 and Imaris V4.0 (Applied Precision, Issaquah, Washington, United States). The two oligonucleotide probes were specific to the homologous regions of the 16S rRNA and were labeled with different fluorescent dyes, enabling visualization of both symbionts within the same preparations.

## Supporting Information

Table S1 Predicted Protein Coding Genes in the *Baumannia* Genome
Predicted functions and role categories are shown.
(659 KB DOC)

Table S2 Predicted Protein Coding Genes in the *Sulcia* Genome
Predicted functions and role categories are shown.
(206 KB DOC)

### Accession Numbers

The genome sequence data have been submitted to multiple sequence databases. All sequence traces have been submitted to the National Center for Biotechnology Information (NCBI) ( http://www.ncbi.nlm.nih.gov) Trace Archive and are available at ftp://ftp.ncbi.nih.gov/pub/TraceDB/baumannia_cicadellinicola. The GenBank ( http://www.ncbi.nlm.nih.gov/Genbank) closed, annotated genome accession number for *Baumannia* is CP000238 and annotated data accession number for *Sulcia* is AANL00000000. The mapping of the traces to the closed genome of *Baumannia* is in the NCBI Assembly Archive ( http://www.ncbi.nlm.nih.gov/Traces/assembly/assmbrowser.cgi)with number pending.

The Institute for Genomic Research (TIGR) accession numbers available in GenBank accession number CP000238 are EnzymeII ^Man^ complex (BCI_0449–0451), phosphotransferase system Enzyme I (BCI_0070), histidyl phosphorylatable protein PtsH (BCI_0069), *mqo* type malate dehydrogenase (BCI_0001), NADH dehydrogenase I (BCI_0369–0381), cytochrome *o* oxidase (BCI_0267–0269), ATP synthase (BCI_0140–0147), glyceraldehyde 3-phosphate dehydrogenase (GapA) (BCI_0443), general amino acid ABC transporter (BCI_0250, BCI_0207–0208), arginine/lysine ABC transporter (BCI_0323–0326), lysine permease (BCI_0393), proton/sodium-glutamate symport protein (BCI_0108), and aspartate ammonia-lyase AspA (BCI_0593),

## Abbreviations

CDS: protein-coding gene
LPS: lipopolysaccharide
pI: isoelectric point
SNP: single nucleotide polymorphism
