# Supplementary material for: Metabolic Complementarity and Genomics of the Dual Bacterial Symbiosis of Sharpshooters
Source: PLoS Biol. 2006 Jun 6;4(6):e188. doi: 10.1371/journal.pbio.0040188 (PMC1472245; doi:10.1371/journal.pbio.0040188)
Supplement: Supplementary file 1 [file pbio.0040188.st001.doc]

Supplemental Table 1: Predicted protein coding genes in the *Baumannia cicadellinicola* genome. Predicted functions and role categories are shown.

| **Accession** | **Gene Symbol** | **Gene Position** | **Gene Name** |
| --- | --- | --- | --- |
| Amino acid biosynthesis;Aspartate family | | | |
| BCI_0017 | metE | 22990-25284 | 5-methyltetrahydropteroyltriglutamate--homocysteine S-methyltransferase |
| BCI_0019 | metA | 28062-27142 | homoserine O-succinyltransferase |
| BCI_0163 | metB | 181851-180688 | O-succinylhomoserine (thiol)-lyase |
| BCI_0407 | aspC | 421467-420277 | aspartate aminotransferase |
| BCI_0540 | metC | 563933-562743 | cystathionine beta-lyase |
| Amino acid biosynthesis;Histidine family | | | |
| BCI_0398 | hisI | 412513-411890 | Phosphoribosyl-AMP cyclohydrolase |
| BCI_0399 | hisF | 413283-412507 | imidazole glycerol phosphate synthase subunit |
| BCI_0400 | hisA | 414011-413265 | phosphoribosylformimino-5-aminoimidazole carboxamide ribotide isomerase |
| BCI_0401 | hisH | 414607-414014 | imidazole glycerol phosphate synthase, glutamine amidotransferase subunit |
| BCI_0402 | hisB | 415674-414604 | histidinol-phosphatase/imidazoleglycerol-phosphate dehydratase |
| BCI_0403 | hisC | 416762-415680 | histidinol-phosphate aminotransferase |
| BCI_0404 | hisD | 418072-416759 | histidinol dehydrogenase |
| BCI_0405 | hisG | 418975-418076 | ATP phosphoribosyltransferase |
| Amino acid biosynthesis;Serine family | | | |
| BCI_0004 | glyA | 6569-7822 | serine hydroxymethyltransferase |
| BCI_0006 | iscS | 8964-10118 | Cysteine desulfurase (ThiI transpersulfidase) (NifSprotein homolog) |
| BCI_0068 | cysK | 80667-81632 | cysteine synthase A |
| BCI_0172 | cysE | 191228-190413 | serine acetyltransferase |
| Biosynthesis of cofactors, prosthetic groups, and carriers;Biotin | | | |
| BCI_0245 | bioA | 261262-259961 | adenosylmethionine-8-amino-7-oxononanoate aminotransferase |
| BCI_0246 | bioB | 261370-262398 | biotin synthase |
| BCI_0247 | bioF | 262395-263573 | KAPA-synthetase |
| BCI_0248 | bioC | 263560-264321 | biotin biosynthesis protein BioC |
| BCI_0249 | bioD | 264314-264991 | dethiobiotin synthase |
| BCI_0485 | bioH | 496688-497449 | bioH protein |
| Biosynthesis of cofactors, prosthetic groups, and carriers;Folic acid | | | |
| BCI_0023 | aroB | 33922-32843 | 3-dehydroquinate synthase |
| BCI_0024 | aroK | 34448-33927 | shikimate kinase |
| BCI_0050 | aroQ | 59746-60249 | 3-dehydroquinate dehydratase, type II |
| BCI_0122 | folD | 136286-135429 | 5,10-methylene-tetrahydrofolate dehydrogenase |
| BCI_0162 | metF | 180507-179599 | 5,10-methylenetetrahydrofolate reductase |
| BCI_0191 | aroH | 203302-204297 | phospho-2-dehydro-3-deoxyheptonate aldolase |
| BCI_0229 | folK | 244506-245009 | 7, 8-dihydro-6-hydroxymethylpterin-pyrophosphokina se |
| BCI_0253 | aroA | 268182-269468 | 3-phosphoshikimate 1-carboxyvinyltransferase |
| BCI_0361 | aroC | 367046-368113 | chorismate synthase |
| BCI_0366 | folC | 372641-373921 | dihydrofolate:folylpolyglutamate synthetase |
| BCI_0394 | folE | 407146-407808 | GTP cyclohydrolase I |
| BCI_0418 | aroE | 430617-431381 | dehydroshikimate reductase |
| BCI_0432 | pabC | 447531-446728 | 4-amino-4-deoxychorismate lyase |
| BCI_0453 | pabB | 469080-467722 | para-aminobenzoate synthase, component I |
| BCI_0525 |  | 545576-544710 | GTP cyclohydrolase, putative |
| BCI_0549 | pabA | 577817-577227 | Para-aminobenzoate synthase glutamine amidotransferase component II |
| BCI_0561 | folA | 593304-593801 | dihydrofolate reductase |
| BCI_0620 | folB | 651651-652016 | dihydroneopterin aldolase |
| BCI_0637 | folP | 670808-669942 | dihydropteroate synthase |
| BCI_0648 |  | 681777-681169 | 5-formyltetrahydrofolate cyclo-ligase family protein |
| Biosynthesis of cofactors, prosthetic groups, and carriers;Glutathione and analogs | | | |
| BCI_0201 | gshA | 214122-212551 | glutamate--cysteine ligase |
| BCI_0482 | gshB | 495253-494300 | glutathione synthetase |
| Biosynthesis of cofactors, prosthetic groups, and carriers;Heme, porphyrin, and cobalamin | | | |
| BCI_0061 | hemC | 72229-73164 | porphobilinogen deaminase |
| BCI_0062 | hemD | 73172-73912 | uroporphyrinogen-III synthase |
| BCI_0215 | cysG | 229795-228407 | siroheme synthase |
| BCI_0270 | cyoE | 287236-288102 | protoheme IX farnesyltransferase |
| BCI_0479 | hemN | 493285-492128 | oxygen-independent coproporphyrinogen III oxidase, putative |
| Biosynthesis of cofactors, prosthetic groups, and carriers;Lipoate | | | |
| BCI_0238 | lipA | 255311-256195 | lipoic acid synthetase |
| Biosynthesis of cofactors, prosthetic groups, and carriers;Pantothenate and coenzyme A | | | |
| BCI_0176 | coaD | 194714-195205 | pantetheine-phosphate adenylyltransferase |
| BCI_0183 | dfp | 199922-198708 | phosphopantothenoylcysteine decarboxylase/phosphopantothenate--cysteine ligase |
| BCI_0230 | panB | 245066-245863 | 3-methyl-2-oxobutanoate hydroxymethyltransferase |
| BCI_0231 | panC | 245882-246739 | pantoate--beta-alanine ligase |
| BCI_0272 | panE | 289569-290438 | 2-dehydropantoate 2-reductase |
| BCI_0359 | coaA | 365096-366034 | pantothenate kinase |
| BCI_0514 | coaE | 529589-530200 | dephospho-CoA kinase |
| Biosynthesis of cofactors, prosthetic groups, and carriers;Pyridine nucleotides | | | |
| BCI_0102 | nadA | 111992-113038 | quinolinate synthetase complex, A subunit |
| BCI_0234 | nadD | 250770-251423 | nicotinate (nicotinamide) nucleotide adenylyltransferase |
| BCI_0288 | nadE | 308545-309399 | NAD+ synthetase |
| BCI_0513 | nadC | 528651-529538 | nicotinate-nucleotide pyrophosphorylase |
| BCI_0611 | nadK | 642955-642050 | ATP-dependent NAD Kinase |
| BCI_0614 | nadB | 646057-644408 | L-aspartate oxidase |
| Biosynthesis of cofactors, prosthetic groups, and carriers;Pyridoxine | | | |
| BCI_0082 | pdxH | 98240-97590 | pyridoxamine 5'-phosphate oxidase |
| BCI_0252 | serC | 266996-268087 | Phosphoserine aminotransferase (PSAT) |
| BCI_0279 | pdxJ | 298507-297776 | pyridoxal phosphate biosynthetic protein PdxJ |
| BCI_0363 | pdxB | 369658-370788 | erythronate-4-phosphate dehydrogenase |
| BCI_0564 | pdxA | 596711-595707 | 4-hydroxythreonine-4-phosphate dehydrogenase |
| Biosynthesis of cofactors, prosthetic groups, and carriers;Riboflavin, FMN, and FAD | | | |
| BCI_0087 | ribE | 101196-100573 | riboflavin synthase, alpha subunit |
| BCI_0300 | ribA | 320416-321015 | GTP cyclohydrolase II |
| BCI_0555 | ribF | 583424-584407 | riboflavin biosynthesis protein RibF |
| BCI_0601 | ribD | 630847-631983 | riboflavin biosynthesis protein RibD |
| BCI_0602 | ribH | 632053-632571 | 6,7-dimethyl-8-ribityllumazine synthase |
| BCI_0623 | ribB | 655165-655800 | 3,4-dihydroxy-2-butanone 4-phosphate synthase |
| Biosynthesis of cofactors, prosthetic groups, and carriers;Thiamine | | | |
| BCI_0041 | thiC | 51357-53225 | Thiamine biosynthesis protein thiC |
| BCI_0042 | thiE | 53212-53856 | thiamine-phosphate pyrophosphorylase |
| BCI_0043 | thiF | 53849-54622 | 4-methyl-5-(beta-hydroxyethyl)thiazole monophosphate synthesis protein |
| BCI_0044 | thiS | 54622-54822 | thiamine biosynthesis protein ThiS |
| BCI_0045 | thiG | 54826-55605 | thiamin biosynthesis protein ThiG |
| BCI_0046 | thiH | 55602-56750 | thiH protein |
| BCI_0273 | thiI | 290748-290452 | Thiamine biosynthesis protein thiI |
| BCI_0275 | dxs | 292085-293956 | 1-deoxy-D-xylulose-5-phosphate synthase |
| BCI_0604 | thiL | 633099-634076 | thiamine-monophosphate kinase |
| BCI_0649 | thiD | 682739-681936 | phosphomethylpyrimidine kinase |
| Biosynthesis of cofactors, prosthetic groups, and carriers;Other | | | |
| BCI_0008 | ispG | 10933-12054 | 1-hydroxy-2-methyl-2-(E)-butenyl 4-diphosphate synthase |
| BCI_0210 | ispF | 224330-223854 | 2C-methyl-D-erythritol 2,4-cyclodiphosphate synthase |
| BCI_0211 | ispD | 225065-224340 | 2-C-methyl-D-erythritol 4-phosphate cytidylyltransferase |
| BCI_0219 | PTPS | 234503-234892 | 6-pyruvoyl-tetrahydropterin synthase |
| BCI_0274 | ispA | 291132-292046 | geranyltranstransferase |
| BCI_0292 | ispE | 312361-313236 | 4-diphosphocytidyl-2C-methyl-D-erythritol kinase |
| BCI_0461 | sufE | 475515-475081 | SufE protein |
| BCI_0462 | sufS | 476754-475531 | cysteine desulfurase |
| BCI_0463 | sufD | 478049-476751 | FeS assembly protein SufD |
| BCI_0464 | sufC | 478770-478030 | FeS assembly ATPase SufC |
| BCI_0465 | sufB | 480320-478845 | FeS assembly protein SufB |
| BCI_0466 | sufA | 480694-480335 | SufA protein |
| BCI_0531 | dxr | 550012-551208 | 1-deoxy-D-xylulose 5-phosphate reductoisomerase |
| BCI_0532 | uppS | 551338-552099 | undecaprenyl diphosphate synthase |
| BCI_0558 | ispH | 587767-588708 | 4-hydroxy-3-methylbut-2-enyl diphosphate reductase |
| Cell envelope;Biosynthesis and degradation of murein sacculus and peptidoglycan | | | |
| BCI_0028 | murA | 38522-39778 | UDP-N-acetylglucosamine 1-carboxyvinyltransferase |
| BCI_0047 | mreD | 57287-56811 | rod shape-determining protein MreD |
| BCI_0048 | mreC | 58312-57446 | rod shape-determining protein MreC |
| BCI_0049 | mreB | 59390-58347 | Rod shape-determining protein mreB |
| BCI_0125 | dapF | 137965-138789 | diaminopimelate epimerase |
| BCI_0139 | glmU | 155609-154200 | UDP-N-acetylglucosamine pyrophosphorylase |
| BCI_0153 | murI | 169657-170481 | glutamate racemase |
| BCI_0226 | mrcB | 241998-241885 | peptidoglycan synthetase; penicillin-binding protein 1B, degenerate |
| BCI_0235 | mrdA | 251494-253359 | peptidoglycan synthetase, penicillin-binding protein 2 |
| BCI_0236 | mrdB | 253373-254464 | rod shape-determining protein RodA |
| BCI_0388 | ddlA | 402168-403295 | D-ala D-ala ligase |
| BCI_0477 | mltC | 491704-490682 | membrane-bound lytic murein transglycosylase C precursor (murein hydrolase C) |
| BCI_0504 | murB | 515660-516688 | UDP-N-acetylenolpyruvoylglucosamine reductase |
| BCI_0518 | murC | 536735-535263 | UDP-N-acetylmuramate--alanine ligase |
| BCI_0519 | murG | 537883-536807 | UDP-N-acetylglucosamine--N-acetylmuramyl-(pentapeptide) pyrophosphoryl-undecaprenol N-acetylglucosamine transferase |
| BCI_0520 | murD | 539540-538200 | UDP-N-acetylmuramoylalanine--D-glutamate ligase |
| BCI_0521 | mraY | 540624-539542 | phospho-N-acetylmuramoyl-pentapeptide-transferase |
| BCI_0522 | murF | 541979-540618 | UDP-N-acetylmuramoyl-tripeptide--D-alanyl-D-alanine ligase |
| BCI_0523 | murE | 543469-541982 | UDP-N-acetylmuramoylalanyl-D-glutamate--2,6-diaminopimelate ligase |
| BCI_0585 |  | 615036-613888 | N-acetylmuramoyl-l-alanine amidase II |
| BCI_0641 | dacB | 674444-675871 | D-alanyl-D-alanine carboxypeptidase/D-alanyl-D-alanine-endopeptidase |
| Cell envelope;Other | | | |
| BCI_0053 |  | 62973-62338 | membrane protein, putative |
| BCI_0089 |  | 101592-101936 | major outer membrane lipoprotein STY1745 -related protein |
| BCI_0099 |  | 109296-110596 | TolB protein, degenerate |
| BCI_0100 | pal | 110637-111272 | peptidoglycan-associated lipoprotein precursor |
| BCI_0136 |  | 149342-150943 | Inner membrane protein oxaA |
| BCI_0391 | spr | 404461-403874 | Lipoprotein spr precursor |
| BCI_0535 |  | 554367-556748 | outer membrane protein precursor yaeT |
| BCI_0536 |  | 556809-557324 | automembrane protein H |
| BCI_0606 |  | 634645-636420 | outer membrane protein |
| Cellular processes;Adaptations to atypical conditions | | | |
| BCI_0261 | cspE | 275772-275503 | Cold shock-like protein cspE |
| Cellular processes;Cell division | | | |
| BCI_0056 | ftsY | 67591-68655 | cell division membrane protein |
| BCI_0057 | ftsE | 68668-69309 | cell division ATP-binding protein |
| BCI_0058 | ftsX | 69329-70279 | cell division protein |
| BCI_0067 | zipA | 80431-79622 | cell division protein ZipA |
| BCI_0148 | gidA | 164129-162234 | glucose inhibited division protein A |
| BCI_0320 | ftsK | 345943-343943 | DNA translocase ftsK |
| BCI_0444 | minC | 459428-460123 | septum site-determining protein MinC |
| BCI_0445 | minD | 460140-460952 | septum site-determining protein MinD |
| BCI_0446 | minE | 460956-461237 | cell division topological specificity factor MinE |
| BCI_0517 | ftsZ | 534951-533779 | cell division protein FtsZ |
| BCI_0638 | ftsH | 672809-670917 | integral membrane peptidase, cell division protein |
| BCI_0639 | ftsJ | 673488-672859 | FtsJ-like methyltransferase |
| Cellular processes;Detoxification | | | |
| BCI_0483 | sodA | 495935-495312 | Superoxide dismutase [Mn] |
| BCI_0595 | tsaA | 624992-624390 | alkyl hydroperoxide reductase, small subunit (antioxidant) |
| Cellular processes;Toxin production and resistance | | | |
| BCI_0452 |  | 466223-467557 | integral membrane protein / hemolysin |
| BCI_0621 |  | 652155-652982 | undecaprenol kinase, putative |
| Cellular processes;Other | | | |
| BCI_0014 |  | 20049-20594 | ADP compounds hydrolase NudE |
| BCI_0280 | era | 299546-298641 | GTP-binding protein Era |
| BCI_0542 | nudH | 565073-565552 | (Di)nucleoside polyphosphate hydrolase (Ap5Apyrophosphatase) |
| Central intermediary metabolism;Amino sugars | | | |
| BCI_0138 | glmS | 154157-152319 | glucosamine--fructose-6-phosphate aminotransferase, isomerizing |
| BCI_0636 | glmM | 669945-668608 | phosphoglucosamine mutase |
| Central intermediary metabolism;Phosphorus compounds | | | |
| BCI_0074 | ppk | 87323-89410 | polyphosphate kinase |
| BCI_0608 | ppa | 639914-639381 | inorganic pyrophosphatase |
| Central intermediary metabolism;Sulfur metabolism | | | |
| BCI_0212 | cysC | 226003-225380 | adenylylsulfate kinase |
| BCI_0213 | cysN | 227442-226000 | ATP-sulfurylase, subunit 1 |
| BCI_0214 | cysD | 228363-227455 | ATP sulfurylase D subunit |
| BCI_0216 | cysH | 230700-229963 | phosophoadenylyl-sulfate reductase |
| BCI_0217 | cysI | 232414-230714 | sulfite reductase (NADPH) hemoprotein, beta subunit |
| BCI_0218 | cysJ | 234201-232414 | sulfite reductase [NADPH] flavoprotein, alpha chain |
| BCI_0413 | dsvC | 428023-427532 | dissimilatory sulfite reductase, gamma subunit |
| BCI_0489 | dsrE | 498341-498712 | Intracellular sulfur reduction protein dsrE |
| BCI_0490 | dsrF | 498724-499083 | introcellular reduction protein dsrF |
| BCI_0491 | dsrH | 499093-499380 | intracellular sulfur oxidation protein dsrH |
| BCI_0570 | cysQ | 600622-599879 | 3'(2'),5'-bisphosphate nucleotidase |
| Central intermediary metabolism;Other | | | |
| BCI_0005 | suhB | 8661-7855 | Inositol-1-monophosphatase (IMPase) |
| BCI_0015 | metK | 20778-21935 | S-adenosylmethionine synthetase |
| BCI_0224 | pfs | 239974-240663 | MTA/SAH nucleosidase |
| DNA metabolism;Chromosome-associated proteins | | | |
| BCI_0040 | HU | 50584-50300 | DNA-binding protein HU-alpha (NS2) (HU-2) |
| BCI_0306 | hns | 328633-329043 | H-NS protein |
| DNA metabolism;DNA replication, recombination, and repair | | | |
| BCI_0018 | rmuC | 25559-27043 | DNA recombination protein rmuC homolog |
| BCI_0066 | ligA | 79558-77543 | DNA ligase, NAD-dependent |
| BCI_0107 | ssb | 118782-118285 | single-strand binding protein |
| BCI_0112 | recG | 123267-125366 | ATP-dependent DNA helicase RecG |
| BCI_0114 | polA | 127267-126347 | DNA polymerase I |
| BCI_0115 | dnaX | 127463-128617 | DNA polymerase III, subunits gamma and tau |
| BCI_0127 | uvrD | 139673-141613 | DNA helicase II |
| BCI_0131 | gyrB | 146565-144157 | DNA gyrase, B subunit |
| BCI_0132 | dnaN | 147835-146723 | DNA polymerase III, beta subunit |
| BCI_0150 | recJ | 165305-167032 | single-stranded-DNA-specific exonuclease RecJ |
| BCI_0166 | priA | 182884-184998 | primosomal protein N' |
| BCI_0177 | mutM | 196011-195202 | formamidopyrimidine-DNA glycosylase |
| BCI_0206 | recA | 218989-217937 | recA protein |
| BCI_0209 | mutS | 221028-223595 | DNA mismatch repair protein MutS |
| BCI_0233 | holA | 249740-250777 | DNA-directed DNA polymerase delta chain |
| BCI_0286 | holC | 306646-306182 | DNA polymerase III, chi subunit |
| BCI_0302 | topA | 324854-322281 | DNA topoisomerase I |
| BCI_0312 | ruvB | 337045-336038 | Holliday junction DNA helicase RuvB |
| BCI_0313 | ruvA | 337644-337054 | Holliday junction DNA helicase RuvA |
| BCI_0314 | ruvC | 338207-337686 | crossover junction endodeoxyribonuclease RuvC |
| BCI_0387 | gyrA | 399296-401839 | DNA gyrase, A subunit |
| BCI_0430 | holB | 446032-445034 | DNA polymerase III, delta' subunit |
| BCI_0473 | dnaQ | 488373-487651 | DNA polymerase III, epsilon subunit |
| BCI_0538 | dnaE | 558254-561745 | DNA polymerase III, alpha subunit |
| BCI_0541 | mutH | 564241-564915 | DNA mismatch repair protein |
| BCI_0545 | recC | 567514-570810 | exodeoxyribonuclease V, gamma subunit |
| BCI_0546 | recB | 570854-574405 | exodeoxyribonuclease V, beta subunit |
| BCI_0547 | recD | 574453-576312 | exodeoxyribonuclease V, alpha subunit |
| BCI_0550 | dnaB | 577977-579389 | replicative DNA helicase |
| BCI_0573 | priN | 601765-601412 | primosomal replication protein-N |
| BCI_0584 | mutL | 613811-612003 | DNA mismatch repair protein MutL |
| BCI_0613 | ung | 644354-643686 | uracil-DNA glycosylase |
| BCI_0617 | dnaG | 650074-648287 | DNA primase |
| BCI_0625 | holD | 657367-656960 | DNA-directed DNA polymerase psi chain |
| DNA metabolism;Degradation of DNA | | | |
| BCI_0039 | nuc | 50198-49665 | endonuclease |
| BCI_0392 | nfo | 405371-404493 | endonuclease IV |
| BCI_0429 |  | 444989-444189 | deoxyribonuclease |
| BCI_0476 |  | 490600-489764 | Endonuclease/Exonuclease/phosphatase family protein |
| Energy metabolism;ATP-proton motive force interconversion | | | |
| BCI_0140 | atpC | 156085-155663 | ATP synthase F1, epsilon subunit |
| BCI_0141 | atpD | 157472-156099 | ATP synthase F1, beta subunit |
| BCI_0142 | atpG | 158381-157518 | ATP synthase F1, gamma subunit |
| BCI_0143 | atpA | 159969-158428 | ATP synthase F1, alpha subunit |
| BCI_0144 | atpH | 160524-159991 | ATP synthase F1, delta subunit |
| BCI_0145 | atpF | 161017-160547 | ATP synthase F0, B subunit |
| BCI_0146 | atpE | 161288-161046 | atp synthase C chain PA5559 |
| BCI_0147 | atpB | 162146-161325 | ATP synthase F0, A subunit |
| Energy metabolism;Amino acids and amines | | | |
| BCI_0149 | gcvT | 165153-164176 | Glycine cleavage T-protein |
| BCI_0593 | aspA | 622569-623987 | aspartate ammonia-lyase |
| Energy metabolism;Electron transport | | | |
| BCI_0060 | ubiB | 71375-72094 | ferrisiderophore reductase |
| BCI_0080 | rnfB | 95990-96178 | ferredoxin II, 4Fe-4S bacterial type |
| BCI_0095 | fldA | 104908-104378 | flavodoxin |
| BCI_0189 | trxA | 202507-202181 | thioredoxin 1 |
| BCI_0267 | cyoA | 283344-284258 | cytochrome O ubiquinol oxidase chain II |
| BCI_0268 | cyoB | 284289-286286 | Cytochrome O ubiquinol oxidase subunit I |
| BCI_0269 | cyoC | 286273-286884 | Cytochrome c oxidase subunit III |
| BCI_0321 | trxB | 346137-347096 | thioredoxin-disulfide reductase |
| BCI_0369 | nuoN | 377832-376378 | NADH2 dehydrogenase (ubiquinone) chain N |
| BCI_0370 | nuoM | 379380-377857 | NADH-quinone oxidoreductase chain M |
| BCI_0371 | nuoL | 381295-379430 | NADH2 dehydrogenase (ubiquinone) chain L |
| BCI_0372 | nuoK | 381594-381292 | NADH2 dehydrogenase (ubiquinone) chain K |
| BCI_0373 | nuoJ | 382139-381642 | NADH2 dehydrogenase (ubiquinone) chain J |
| BCI_0374 | nuoI | 382705-382163 | NADH2 dehydrogenase (ubiquinone) chain I (EC 1.6.5.3) |
| BCI_0375 | nuoH | 383694-382729 | NADH-quinone oxidoreductase chain H (NADH dehydrogenaseI, chain H) |
| BCI_0376 | nuoG | 386432-383703 | NADH-quinone oxidoreductase, chain G |
| BCI_0377 | nuoF | 387848-386514 | NADH-quinone oxidoreductase, F subunit |
| BCI_0378 | nuoE | 388348-387845 | NADH dehydrogenase I chain E |
| BCI_0379 | nuoCD | 390138-388351 | NADH2 dehydrogenase (ubiquinone) chain C/D |
| BCI_0380 | nuoB | 390921-390247 | NADH dehydrogenase I chain B (NADH-ubiquinone oxidoreductase chain 2) |
| BCI_0381 | nuoA | 391371-390985 | NADH-quinone oxidoreductase chain A (NADH dehydrogenaseI, chain A) |
| BCI_0384 |  | 395295-395029 | iron-sulfur cluster binding protien |
| BCI_0419 |  | 434917-431426 | Pyruvate-flavodoxin oxidoreductase |
| Energy metabolism;Fermentation | | | |
| BCI_0382 | ackA | 391619-392821 | acetate kinase |
| BCI_0383 | pta | 392875-395010 | phosphate acetyltransferase |
| Energy metabolism;Glycolysis/gluconeogenesis | | | |
| BCI_0012 | pgi | 16150-17811 | glucose-6-phosphate isomerase |
| BCI_0013 | pckA | 19740-18124 | phosphoenolpyruvate carboxykinase (ATP) |
| BCI_0169 | tpiA | 187372-188142 | triosephosphate isomerase |
| BCI_0171 | pfkA | 190166-189204 | 6-Phosphofructokinase I |
| BCI_0221 | eno | 236928-235627 | enolase |
| BCI_0251 | gpmA | 266276-266989 | phosphoglycerate mutase 1 |
| BCI_0310 | pykA | 332644-334095 | pyruvate kinase |
| BCI_0443 | gapA | 458115-459185 | glyceraldehyde-3-phosphate dehydrogenase, type I |
| BCI_0475 | gloB | 488969-489742 | hydroxyacylglutathione hydrolase |
| BCI_0645 | pgk | 677985-679148 | phosphoglycerate kinase |
| BCI_0646 | fbaA | 679227-680303 | fructose-bisphosphate aldolase, class II |
| Energy metabolism;Pentose phosphate pathway | | | |
| BCI_0022 | rpe | 32749-32054 | ribulose-phosphate 3-epimerase |
| BCI_0075 | tktA | 91435-89438 | transketolase |
| BCI_0076 | talB | 92409-91456 | transaldolase |
| BCI_0244 | pgl | 259068-259949 | 6-phosphogluconolactonase |
| BCI_0309 | zwf | 332223-330745 | glucose-6-phosphate 1-dehydrogenase |
| BCI_0397 | gnd | 411687-410281 | 6-phosphogluconate dehydrogenase, decarboxylating |
| BCI_0647 | rpiA | 680445-681116 | ribose 5-phosphate isomerase |
| Energy metabolism;Pyruvate dehydrogenase | | | |
| BCI_0510 | lpdA | 524268-522856 | pyruvate dehydrogenase complex, E3 component, dihydrolipoamide dehydrogenase |
| BCI_0511 | aceF | 525403-524327 | pyruvate dehydrogenase complex, E2 component, dihydrolipoamide acetyltransferase |
| BCI_0512 | aceE | 528296-525627 | pyruvate dehydrogenase (lipoamide) E1 component |
| Energy metabolism;TCA cycle | | | |
| BCI_0001 | mqo | 197-1720 | malate:quinone-oxidoreductase |
| BCI_0097 | sucC | 106172-107353 | Succinyl-CoA synthetase beta chain (SCS-beta) |
| BCI_0098 | sucD | 107341-108225 | succinate-CoA ligase (ADP-forming) alpha chain |
| BCI_0420 | fumC | 436338-434947 | fumarate hydratase, class II |
| Fatty acid and phospholipid metabolism;Biosynthesis | | | |
| BCI_0020 | plsB | 30780-28315 | glycerol-3-phosphate acyltransferase |
| BCI_0025 | pssA | 36096-34735 | CDP-diacylglycerol--serine O-phosphatidyltransferase |
| BCI_0051 | accB | 60271-60723 | acetyl-CoA carboxylase, biotin carboxyl carrier protein |
| BCI_0052 | accC | 60732-62081 | acetyl-CoA carboxylase, biotin carboxylase |
| BCI_0173 | gpsA | 192301-191279 | Glycerol-3-phosphate dehydrogenase |
| BCI_0240 | pgsA | 257860-258423 | CDP-diacylglycerol--glycerol-3-phosphate 3-phosphatidyltransferase |
| BCI_0278 | acpS | 297776-297336 | holo-(acyl-carrier-protein) synthase |
| BCI_0296 | fabI | 316116-316904 | enoyl-[acyl-carrier-protein] reductase STY1352 |
| BCI_0305 | cls | 326727-328178 | cardiolipin synthase |
| BCI_0362 | fabB | 368364-369584 | 3-oxoacyl-[acyl-carrier-protein] synthase |
| BCI_0365 | accD | 371711-372598 | acetyl-CoA carboxylase, carboxyl transferase, beta subunit |
| BCI_0410 | fabA | 425041-424520 | beta-hydroxyacyl-(acyl-carrier-protein) dehydratase FabA |
| BCI_0433 | acpP | 447865-447632 | acyl carrier protein |
| BCI_0434 | fabG | 448755-448021 | 3-oxoacyl-(acyl-carrier-protein) reductase |
| BCI_0435 | fabD | 449701-448739 | malonyl CoA-acyl carrier protein transacylase |
| BCI_0436 | fabH | 450674-449721 | 3-oxoacyl-[acyl-carrier-protein] synthase III |
| BCI_0533 | cdsA | 552109-552963 | phosphatidate cytidylyltransferase |
| BCI_0537 | fabZ | 557580-558029 | beta-hydroxyacyl-(acyl-carrier-protein) dehydratase FabZ |
| BCI_0539 | accA | 561762-562721 | acetyl-CoA carboxylase, carboxyl transferase, alpha subunit |
| BCI_0587 | psd | 616404-617282 | phosphatidylserine decarboxylase |
| BCI_0605 | pgpA | 634084-634590 | phosphatidylglycerophosphatase |
| BCI_0624 | plsC | 656714-655965 | 1-acyl-sn-glycerol-3-phosphate acyltransferase |
| Protein fate;Degradation of proteins, peptides, and glycopeptides | | | |
| BCI_0029 | degQ | 41209-39827 | serine protease |
| BCI_0054 | prlC | 63370-65457 | Oligopeptidase A |
| BCI_0167 | hslV | 185223-185765 | heat shock protein |
| BCI_0175 |  | 193315-194442 | peptidase, putative |
| BCI_0263 | lon | 279098-276744 | ATP-dependent protease La |
| BCI_0264 | clpX | 280572-279307 | ATP-dependent Clp protease, ATP-binding subunit ClpX |
| BCI_0265 | clpP | 281326-280709 | ATP-dependent Clp protease, proteolytic subunit ClpP |
| BCI_0287 | pepA | 308172-306670 | cytosol aminopeptidase (leucine aminopeptidase) |
| BCI_0411 | lon | 426671-425088 | Lon protease |
| BCI_0442 | sppA | 456213-458069 | signal peptide peptidase SppA, 67K type |
| BCI_0447 |  | 461956-461258 | glycoprotease family protein |
| BCI_0534 |  | 552983-554362 | membrane-associated zinc metalloprotease, putative |
| BCI_0578 | hflC | 607890-606889 | HflC protein |
| BCI_0579 | hflK | 609057-607897 | component of modulator for protease specific for FtsH phage lambda cII repressor |
| BCI_0619 | gcp | 650584-651609 | O-sialoglycoprotein endopeptidase (glycoprotease) |
| Protein fate;Protein and peptide secretion and trafficking | | | |
| BCI_0199 | ffh | 212249-210900 | signal recognition particle protein |
| BCI_0282 | lepB | 301201-300266 | signal peptidase I |
| BCI_0291 | lolB | 311717-312364 | Outer membrane lipoprotein LolB |
| BCI_0319 | lolA | 343841-343215 | outer membrane lipoprotein carrier protein LolA |
| BCI_0348 | secY | 360023-361357 | Preprotein translocase secY subunit |
| BCI_0424 | lolE | 441278-440031 | ABC transporter (lipoprotein) (membrane) |
| BCI_0425 | lolD | 441985-441275 | ABC transporter (lipoprotein) (ATP-binding) |
| BCI_0426 |  | 443171-441978 | lipoprotein releasing system, transmembrane protein, LolC/E family |
| BCI_0496 | SecE | 503929-504312 | Preprotein translocase SecE subunit |
| BCI_0515 | secA | 532891-530324 | preprotein translocase, SecA subunit, authentic point mutation |
| BCI_0557 | lspA | 587235-587738 | signal peptidase II |
| BCI_0598 |  | 627436-627768 | Preprotein translocase YajC |
| BCI_0599 | secD | 627802-629640 | protein-export membrane protein SecD |
| BCI_0600 | secF | 629651-630571 | protein-export membrane protein SecF |
| BCI_0635 | secG | 668506-668228 | Protein-export membrane protein secG |
| Protein fate;Protein folding and stabilization | | | |
| BCI_0037 | ibp | 48623-48144 | Small heat shock protein ibp |
| BCI_0117 | htpG | 129408-131303 | heat shock protein HtpG |
| BCI_0168 | hslU | 185769-187109 | heat shock protein HslVU, ATPase subunit HslU |
| BCI_0194 | clpB | 206288-208876 | ATP-dependent chaperone protein ClpB |
| BCI_0257 | dsbA | 273115-272438 | Thiol:disulfide interchange protein dsbA precursor |
| BCI_0266 | tig | 282858-281548 | trigger factor |
| BCI_0307 | htpX | 329964-329089 | heat shock protein, integral membrane protein |
| BCI_0552 | dnaK | 579806-581701 | chaperone protein DnaK |
| BCI_0553 | dnaJ | 581800-582918 | chaperone protein DnaJ |
| BCI_0565 | surA | 597999-596698 | peptidylprolyl cis-trans isomerase SurA |
| BCI_0591 | groEL | 621981-620341 | chaperonin GroEL |
| BCI_0592 | groES | 622316-622023 | chaperonin, 10 kDa |
| BCI_0612 | grpE | 643039-643635 | co-chaperone GrpE |
| Protein fate;Protein modification and repair | | | |
| BCI_0063 | lnt | 75447-73927 | apolipoprotein N-acyltransferase |
| BCI_0237 | lipB | 254592-255275 | lipoate-protein ligase B |
| BCI_0289 | hemK | 310312-309467 | Protein methyltranferase HemK (Protein-glutamine N-methyltransferase) |
| BCI_0360 | prmB | 366096-367031 | protein-(glutamine-N5) methyltransferase |
| BCI_0416 | def | 429705-429202 | peptide deformylase |
| BCI_0505 | birA | 516690-517538 | biotin-[acetyl-CoA-carboxylase] ligase |
| BCI_0526 | map | 546555-545873 | methionine aminopeptidase, type I |
| BCI_0543 | lgt | 565760-566617 | prolipoprotein diacylglyceryl transferase |
| Protein synthesis;Ribosomal proteins | | | |
| BCI_0031 | rplM | 41947-42375 | ribosomal protein L13 |
| BCI_0032 | rpsI | 42395-42793 | ribosomal protein S9 |
| BCI_0133 | rpmH | 148604-148747 | ribosomal protein L34 |
| BCI_0165 | rpmE | 182764-182486 | ribosomal protein L31 |
| BCI_0178 | rpmG | 196239-196072 | ribosomal protein L33 |
| BCI_0179 | rpmB | 196516-196289 | ribosomal protein L28 |
| BCI_0195 | rplS | 209220-208873 | ribosomal protein L19 |
| BCI_0198 | rpsP | 210803-210537 | ribosomal protein S16 |
| BCI_0255 | rpsA | 270340-272016 | ribosomal protein S1 |
| BCI_0327 | rpsJ | 350803-351114 | ribosomal protein S10 |
| BCI_0328 | rplC | 351140-351778 | ribosomal protein L3 |
| BCI_0329 | rplD | 351790-352395 | ribosomal protein L4/L1 family |
| BCI_0330 | rplW | 352392-352694 | ribosomal protein L23 |
| BCI_0331 | rplB | 352714-353532 | ribosomal protein L2 |
| BCI_0332 | rpsS | 353554-353832 | ribosomal protein S19 |
| BCI_0333 | rplV | 353863-354195 | ribosomal protein L22 |
| BCI_0334 | rpsC | 354212-354913 | ribosomal protein S3 |
| BCI_0335 | rplP | 354925-355338 | ribosomal protein L16 |
| BCI_0336 | rpmC | 355335-355526 | ribosomal protein L29 |
| BCI_0337 | rpsQ | 355519-355788 | ribosomal protein S17 VC2587 |
| BCI_0338 | rplN | 355829-356311 | 50S ribosomal protein L14 |
| BCI_0339 | rplX | 356323-356637 | ribosomal protein L24 |
| BCI_0340 | rplE | 356661-357206 | 50S ribosomal protein L5 |
| BCI_0341 | rpsN | 357229-357534 | ribosomal protein S14p |
| BCI_0342 | rpsH | 357567-357965 | ribosomal protein S8 |
| BCI_0343 | rp1F | 357980-358513 | 50S ribosomal subunit protein L6 |
| BCI_0344 | rplR | 358527-358874 | ribosomal protein L18 |
| BCI_0345 | rpsE | 358886-359389 | ribosomal protein S5 |
| BCI_0346 | rpmD | 359393-359575 | ribosomal protein L30 |
| BCI_0347 | rplO | 359576-360007 | ribosomal protein L15 |
| BCI_0349 | rpmJ | 361395-361511 | ribosomal protein L36 |
| BCI_0350 | rpsM | 361537-361893 | ribosomal protein S13p |
| BCI_0351 | rpsK | 361918-362307 | ribosomal protein S11 |
| BCI_0352 | rpsD | 362354-362974 | ribosomal protein S4 |
| BCI_0354 | rplQ | 364042-364428 | ribosomal protein L17 |
| BCI_0390 | rplY | 403741-403457 | Ribosomal L25p family |
| BCI_0438 | rpmF | 451249-451079 | ribosomal protein L32 |
| BCI_0469 | rplT | 484655-484293 | ribosomal protein L20 |
| BCI_0470 | rpmI | 484880-484683 | ribosomal protein L35 |
| BCI_0492 | rpsL | 499438-499812 | ribosomal protein S12 |
| BCI_0493 | rpsG | 499871-500341 | ribosomal protein S7 |
| BCI_0498 | rplK | 504914-505342 | ribosomal protein L11 |
| BCI_0499 | rplA | 505346-506050 | 50S ribosomal protein L1 |
| BCI_0500 | rplJ | 506230-506730 | 50S ribosomal protein L10 |
| BCI_0501 | rplL | 506766-507131 | ribosomal protein L7/L12 |
| BCI_0527 | rpsB | 546860-547558 | ribosomal protein S2 |
| BCI_0554 | rpsT | 583214-582951 | ribosomal protein S20 |
| BCI_0571 | rplI | 601095-600643 | ribosomal protein L9 |
| BCI_0572 | rpsR | 601408-601181 | ribosomal protein S18 |
| BCI_0574 | rpsF | 602146-601769 | 30S ribosomal protein S6 |
| BCI_0618 | rpsU | 650383-650168 | ribosomal protein S21 |
| BCI_0628 | rpsO | 662058-661753 | ribosomal protein S15 |
| BCI_0643 | rpmA | 677197-676943 | ribosomal protein L27 |
| BCI_0644 | rplU | 677523-677212 | ribosomal protein L21 |
| Protein synthesis;Translation factors | | | |
| BCI_0151 | prfB | 167063-168074 | peptide chain release factor 2, programmed frameshift |
| BCI_0290 | prfA | 311409-310309 | peptide chain release factor 1 |
| BCI_0322 | infA | 347211-347429 | translation initiation factor IF-1 |
| BCI_0471 | infC | 485441-484926 | translation initiation factor IF-3 |
| BCI_0494 | fusA | 500446-502563 | translation elongation factor G |
| BCI_0495 | EF-Tu | 502679-503863 | translation elongation factor Tu |
| BCI_0528 | tsf | 547601-548443 | translation elongation factor Ts |
| BCI_0530 | frr | 549301-549858 | ribosome recycling factor |
| BCI_0589 | efp | 619198-618629 | translation elongation factor P |
| BCI_0631 | infB | 666214-663572 | translation initiation factor IF2-2 |
| Protein synthesis;tRNA aminoacylation | | | |
| BCI_0009 | hisS | 12077-13369 | histidyl-tRNA synthetase |
| BCI_0021 | trpS | 31974-30958 | tryptophanyl-tRNA synthetase |
| BCI_0081 | tyrS | 97554-96280 | tyrosyl-tRNA synthetase |
| BCI_0094 | glnS | 102616-104286 | glutaminyl-tRNA synthetase |
| BCI_0121 | cysS | 134027-135415 | cysteinyl-tRNA synthetase |
| BCI_0152 | lysS | 168083-169603 | lysyl-tRNA synthetase |
| BCI_0160 | glyS | 177954-175855 | glycyl-tRNA synthetase, beta subunit |
| BCI_0161 | glyQ | 178868-177960 | glycyl-tRNA synthetase, alpha subunit |
| BCI_0205 | alaS | 217689-215035 | alanyl-tRNA synthetase |
| BCI_0232 | leuS | 246938-249523 | leucyl-tRNA synthetase |
| BCI_0276 | argS | 294026-295762 | arginyl-tRNA synthetase |
| BCI_0285 | valS | 306172-303299 | valyl-tRNA synthetase |
| BCI_0315 | aspS | 340470-338701 | aspartyl-tRNA synthetase |
| BCI_0318 | serS | 343065-341773 | seryl-tRNA synthetase |
| BCI_0396 | metG | 408612-410255 | methionyl-tRNA synthetase |
| BCI_0408 | asnS | 422951-421548 | asparaginyl-tRNA synthetase |
| BCI_0415 | fmt | 429152-428217 | methionyl-tRNA formyltransferase |
| BCI_0456 | gltX | 471677-470277 | glutamyl-tRNA synthetase |
| BCI_0467 | pheT | 483225-480841 | phenylalanyl-tRNA synthetase, beta subunit |
| BCI_0468 | pheS | 484225-483242 | phenylalanyl-tRNA synthetase, alpha subunit |
| BCI_0472 | thrS | 487401-485473 | threonyl-tRNA synthetase |
| BCI_0556 | ileS | 584419-587235 | isoleucyl-tRNA synthetase |
| BCI_0609 | proS | 641747-640074 | prolyl-tRNA synthetase |
| Protein synthesis;tRNA and rRNA base modification | | | |
| BCI_0065 | miaB | 77355-76000 | tRNA-i(6)A37 thiotransferase enzyme MiaB |
| BCI_0103 | tilS | 114455-112995 | tRNA(Ile)-lysidine synthetase |
| BCI_0137 | trmE | 150940-152313 | tRNA modification GTPase TrmE |
| BCI_0193 | rluD | 205190-206149 | Ribosomal large subunit pseudouridine synthase D |
| BCI_0196 | trmD | 209995-209237 | tRNA (guanine-N1)-methyltransferase |
| BCI_0364 | truA | 370796-371599 | tRNA pseudouridine synthase A |
| BCI_0422 | trmU | 437171-438277 | tRNA (5-methylaminomethyl-2-thiouridylate)-methyltransferase |
| BCI_0439 | rluC | 452360-451392 | Ribosomal large subunit pseudouridine synthase C |
| BCI_0563 | ksgA | 595714-594899 | dimethyladenosine transferase |
| BCI_0575 |  | 603046-602300 | RNA methyltransferase, TrmH family, group 3 |
| BCI_0583 | miaA | 611993-611064 | tRNA delta(2)-isopentenylpyrophosphate transferase |
| BCI_0596 | queA | 625179-626240 | S-adenosylmethionine:tRNA ribosyltransferase-isomerase |
| BCI_0597 | tgt | 626302-627414 | queuine tRNA-ribosyltransferase |
| BCI_0622 | cca | 654286-653048 | tRNA nucleotidyltransferase |
| BCI_0629 | truB | 663096-662143 | tRNA pseudouridine synthase B |
| Protein synthesis;Other | | | |
| BCI_0294 | pth | 314337-314918 | peptidyl-tRNA hydrolase |
| Purines, pyrimidines, nucleosides, and nucleotides;2'-Deoxyribonucleotide metabolism | | | |
| BCI_0182 | dut | 198727-198275 | deoxyuridine 5''-triphosphate nucleotidohydrolase |
| BCI_0385 | nrdB | 396427-395297 | ribonucleoside-diphosphate reductase, beta subunit |
| BCI_0386 |  | 398760-396478 | ribonucleoside-diphosphate reductase, alpha subunit |
| BCI_0395 | dcd | 408419-407838 | 2-deoxycytidine 5-triphosphate deaminase |
| BCI_0544 | thyA | 566648-567442 | thymidylate synthase |
| Purines, pyrimidines, nucleosides, and nucleotides;Nucleotide and nucleoside interconversions | | | |
| BCI_0118 | adk | 131400-132041 | Adenylate kinase |
| BCI_0254 | cmk | 269530-270207 | cytidylate kinase |
| Purines, pyrimidines, nucleosides, and nucleotides;Purine ribonucleotide biosynthesis | | | |
| BCI_0002 | purL | 1933-5826 | phosphoribosylformylglycinamidine synthase |
| BCI_0007 | ndk | 10151-10585 | nucleoside-diphosphate kinase |
| BCI_0035 | purH | 45159-46739 | bifunctional purine biosynthesis protein PurH |
| BCI_0036 | purD | 46760-48034 | phosphoribosylamine--glycine ligase |
| BCI_0071 | purC | 84856-84143 | phosphoribosylaminoimidazole-succinocarboxamide synthase |
| BCI_0072 | purM | 85386-86462 | phosphoribosylformylglycinamidine cyclo-ligase |
| BCI_0073 | purN | 86453-87112 | phosphoribosylglycinamide formyltransferase |
| BCI_0110 | gmk | 122086-122700 | Guanylate kinase (GMP kinase) |
| BCI_0119 | purK | 133117-132044 | phosphoribosylaminoimidazole carboxylase, ATPase subunit |
| BCI_0120 | purE | 133623-133114 | phosphoribosylaminoimidazole carboxylase, catalytic subunit |
| BCI_0293 | prsA | 313289-314227 | phosphoribosylpyrophosphate synthetase |
| BCI_0368 | purF | 374840-376258 | amidophosphoribosyltransferase |
| BCI_0423 | purB | 438604-439995 | adenylosuccinate lyase |
| BCI_0577 | purA | 606785-605487 | adenylosuccinate synthetase |
| BCI_0650 | guaA | 684489-682909 | GMP synthetase [glutamine-hydrolyzing] (glutamine amidotransferase) |
| BCI_0651 | guaB | 685996-684539 | inosine-5'-monophosphate dehydrogenase |
| Purines, pyrimidines, nucleosides, and nucleotides;Pyrimidine ribonucleotide biosynthesis | | | |
| BCI_0181 | pyrE | 197558-198220 | orotate phosphoribosyltransferase |
| BCI_0222 | pyrG | 238627-236996 | CTP synthase |
| BCI_0258 | pyrB | 273457-274482 | aspartate carbamoyltransferase |
| BCI_0259 | pyrI | 274496-274966 | aspartate carbamoyltransferase, regulatory subunit |
| BCI_0298 | pyrF | 319750-319001 | orotidine 5'-phosphate decarboxylase |
| BCI_0409 | pyrD | 423377-424387 | dihydroorotate dehydrogenase |
| BCI_0441 | pyrC | 455003-456097 | dihydroorotase, homodimeric type |
| BCI_0529 | pyrH | 548503-549252 | uridylate kinase |
| BCI_0559 | carA | 588858-590003 | carbamoyl-phosphate synthase, small subunit |
| BCI_0560 | carB | 590022-593261 | carbamoyl-phosphate synthase, large subunit |
| Purines, pyrimidines, nucleosides, and nucleotides;Salvage of nucleosides and nucleotides | | | |
| BCI_0431 | tmk | 446673-446035 | thymidylate kinase |
| BCI_0562 | apaH | 594669-593803 | bis(5'-nucleosyl)-tetraphosphatase (symmetrical) |
| BCI_0627 | pnp | 661642-659549 | polynucleotide phosphorylase |
| Regulatory functions;Other | | | |
| BCI_0124 | cyaA | 136971-137886 | adenylate cyclase, degenerate |
| BCI_0204 | csrA | 214853-214668 | carbon storage regulator |
| BCI_0223 | relA | 239625-238739 | GTP pyrophosphokinase, degenerate |
| BCI_0488 |  | 497782-498225 | zinc uptake regulation protein |
| Transcription;DNA-dependent RNA polymerase | | | |
| BCI_0111 | rpoZ | 122773-122970 | DNA-directed RNA polymerase, omega subunit |
| BCI_0353 | rpoA | 362995-363978 | DNA-directed RNA polymerase, alpha subunit |
| BCI_0502 | rpoB | 507305-511327 | DNA-directed RNA polymerase, beta subunit |
| BCI_0503 | rpoC | 511416-515642 | DNA-directed RNA polymerase beta' chain |
| Transcription;Degradation of RNA | | | |
| BCI_0085 | rnt | 99312-99968 | ribonuclease T |
| BCI_0134 | rnpA | 148759-149106 | ribonuclease P protein component |
| BCI_0260 |  | 275012-275407 | endoribonuclease L-PSP, putative |
| BCI_0281 | rnc | 300226-299549 | Ribonuclease III (RNase III) |
| BCI_0297 | rnb | 317057-318994 | exoribonuclease II |
| BCI_0440 | rne | 452878-454647 | ribonuclease E |
| BCI_0474 | rnhA | 488423-488884 | RNase H |
| BCI_0576 | rnr | 605384-603135 | ribonuclease R |
| BCI_0586 | orn | 616028-615474 | oligoribonuclease |
| Transcription;RNA processing | | | |
| BCI_0197 | rimM | 210547-210020 | 16S rRNA processing protein rimM |
| BCI_0228 | pcnB | 243431-244480 | poly(A) polymerase |
| BCI_0630 | rbfA | 663503-663099 | ribosome-binding factor A |
| Transcription;Transcription factors | | | |
| BCI_0016 | metR | 22887-21979 | lysR-family transcription regulatory protein metR |
| BCI_0059 | rpoH | 70342-71202 | RNA polymerase sigma-32 factor |
| BCI_0083 | slyA | 98908-98474 | Transcriptional regulator slyA |
| BCI_0109 | marR | 121414-121839 | MarR family transcription regulator |
| BCI_0301 | cysB | 322032-321058 | Cys regulon transcriptional activator CysB |
| BCI_0548 | crp | 576511-577143 | cAMP-regulatory protein crp |
| BCI_0616 | rpoD | 648205-646367 | RNA polymerase, sigma(70) factor |
| BCI_0640 | greA | 674241-673765 | transcription elongation factor greA (transcript cleavage factor) |
| Transcription;Other | | | |
| BCI_0164 | metJ | 182161-182447 | transcription repressor protein MetJ, authentic frameshift |
| BCI_0188 | rho | 202087-200828 | transcription termination factor Rho |
| BCI_0227 | dksA | 242466-242921 | DnaK suppressor protein DksA |
| BCI_0497 | nusG | 504313-504858 | transcription termination/antitermination factor NusG |
| BCI_0581 | hfq | 610751-610491 | RNA chaperone Hfq |
| BCI_0603 | nusB | 632598-633017 | transcription antitermination factor NusB |
| BCI_0626 | deaD | 659135-657360 | inducible ATP-independent RNA helicase |
| BCI_0632 | nusA | 667728-666241 | transcription pausing L factor |
| Transport and binding proteins;Amino acids, peptides and amines | | | |
| BCI_0108 | gltP | 119468-120774 | proton glutamate symport protein, authentic frameshift |
| BCI_0207 |  | 219476-220258 | amino acid ABC transporter, periplasmic amino acid-binding protein |
| BCI_0208 |  | 220285-220941 | amino acid ABC transport system permease protein |
| BCI_0250 |  | 265455-266216 | amino acid transport ATP-binding protein |
| BCI_0323 | artP | 347605-348333 | arginine transport ATP-binding protein ArtP |
| BCI_0324 | artI | 348354-349082 | arginine 3rd transport system periplasmic binding protein |
| BCI_0325 | artQ | 349079-349774 | arginine 3rd transport system permease protein |
| BCI_0326 | artM | 349796-350467 | arginine transport system permease protein artM |
| BCI_0393 | lysP | 405524-407002 | lysine-specific permease |
| Transport and binding proteins;Anions | | | |
| BCI_0055 | pitA | 67028-65454 | low-affinity inorganic phosphate transport protein |
| BCI_0077 | cysT | 92900-93730 | sulfate transport system permease protein CysT |
| BCI_0078 | cysW | 93739-94581 | Sulfate transport system permease protein cysW |
| BCI_0079 | cysA | 94584-95717 | ATP-binding component of sulfate permease A protein |
| BCI_0170 | sbp | 189133-188144 | periplasmic sulfate-binding protein of sulfate/thiosulfate ABC transporter |
| Transport and binding proteins;Carbohydrates, organic alcohols, and acids | | | |
| BCI_0069 | ptsH | 81806-82063 | Phosphocarrier protein HPr (Histidine-containing protein) |
| BCI_0070 | ptsI | 82116-83831 | phosphoenolpyruvate-protein phosphotransferase |
| BCI_0449 |  | 463897-463058 | PTS system, mannose/fructose/sorbose family, IID component |
| BCI_0450 | manY | 464719-463919 | mannose-specific PTS enzyme IIC |
| BCI_0451 | manX | 465724-464756 | mannose-specific PTS enzyme IIAB |
| Transport and binding proteins;Cations and iron carrying compounds | | | |
| BCI_0104 |  | 116122-114464 | Na+/H+ antiporter |
| BCI_0105 |  | 117356-116502 | cation ABC transporter, permease protein |
| BCI_0106 | troB | 118200-117358 | cation ABC transporter, ATP-binding protein |
| BCI_0128 | corA | 142060-143010 | magnesium and cobalt transport protein CorA |
| BCI_0308 | ftn | 330657-330169 | cytoplasmic ferritin |
| BCI_0311 | mntH | 334515-335864 | NRAMP family Mn2+/Fe2+ transporters |
| Transport and binding proteins;Other | | | |
| BCI_0459 |  | 473538-472021 | drug resistance transporter, EmrB/QacA subfamily |
| BCI_0460 | emrA | 474784-473573 | multidrug resistance protein PA5159 |
| Transport and binding proteins;Unknown substrate | | | |
| BCI_0239 |  | 256452-257660 | transporter, major facilitator family |
| BCI_0271 |  | 288131-289540 | hpothetical major facilitator family transport protein |
| Unknown function;Enzymes of unknown specificity | | | |
| BCI_0003 |  | 5880-6356 | cytidine/deoxycytidylate deaminase family protein |
| BCI_0010 |  | 13482-14615 | PQQ enzyme repeat domain protein |
| BCI_0524 | mraW | 544231-543656 | MraW methylase family |
| Unknown function;General | | | |
| BCI_0011 | engA | 14634-16052 | GTP-binding protein EngA |
| BCI_0026 |  | 36927-36277 | putative DedA-family integral membrane protein |
| BCI_0027 |  | 38197-38454 | BolA/YrbA family protein |
| BCI_0030 |  | 41711-41304 | probable membrane protein YPO3565 |
| BCI_0034 |  | 44258-45016 | ATPase family protein, authentic frameshift |
| BCI_0086 |  | 100367-100008 | glutaredoxin-related protein |
| BCI_0113 |  | 125424-126032 | conserved possible cell division GTP-binding protein |
| BCI_0126 |  | 138902-139618 | haloacid dehalogenase-like hydrolase |
| BCI_0174 |  | 193118-192687 | rhodanese domain protein |
| BCI_0225 |  | 241054-240707 | HesB family protein |
| BCI_0262 | exsB | 276000-276716 | exsB protein |
| BCI_0277 | mviN | 297339-295813 | integral membrane protein MviN |
| BCI_0283 | lepA | 303018-301210 | GTP-binding protein LepA |
| BCI_0295 | ychF | 314968-316038 | GTP-binding protein YchF |
| BCI_0316 |  | 341429-340695 | PHP family protein |
| BCI_0367 | cvpA | 374315-374800 | CvpA family protein |
| BCI_0406 |  | 419621-420250 | metallo-beta-lactamase superfamily protein |
| BCI_0428 |  | 444192-443827 | Hit family protein |
| BCI_0454 |  | 469202-470176 | rhodanese-like domain protein |
| BCI_0484 |  | 496540-495968 | Protein yhgI |
| BCI_0566 | smpA | 598048-598380 | small protein A |
| BCI_0568 | smpB | 598913-599398 | SsrA-binding protein |
| BCI_0580 | hflX | 610383-609100 | GTP-binding protein hflX |
| BCI_0588 |  | 618541-617558 | lysyl-tRNA synthetase homolog GenX |
| BCI_0590 |  | 619232-620251 | YodO family protein |
| BCI_0607 |  | 636417-639365 | probable exported protein YPO3523 |
| BCI_0642 |  | 676891-675881 | GTP1/Obg family protein |
| Hypothetical proteins;Conserved | | | |
| BCI_0033 |  | 43253-43747 | conserved hypothetical protein TIGR00645 |
| BCI_0038 |  | 49642-48740 | conserved hypothetical protein |
| BCI_0064 |  | 75960-75493 | conserved hypothetical protein |
| BCI_0096 |  | 105071-105817 | conserved hypothetical protein TIGR00486 |
| BCI_0101 |  | 111287-111934 | conserved hypothetical protein |
| BCI_0116 |  | 128892-129230 | conserved hypothetical protein TIGR00103 |
| BCI_0130 |  | 143135-144142 | conserved hypothetical protein |
| BCI_0135 |  | 149082-149339 | conserved hypothetical protein TIGR00278 |
| BCI_0180 |  | 197481-196615 | conserved hypothetical protein TIGR00255 |
| BCI_0190 |  | 202969-202631 | conserved hypothetical protein |
| BCI_0192 |  | 205091-204396 | conserved hypothetical protein |
| BCI_0220 |  | 235586-234915 | conserved hypothetical protein |
| BCI_0256 |  | 272212-272376 | conserved hypothetical protein |
| BCI_0299 |  | 320081-319785 | conserved hypothetical protein |
| BCI_0303 |  | 325304-326068 | conserved hypothetical protein YciC |
| BCI_0304 |  | 326263-326565 | conserved hypothetical protein |
| BCI_0412 |  | 427426-427109 | conserved hypothetical protein |
| BCI_0417 |  | 429937-430533 | conserved hypothetical protein |
| BCI_0427 |  | 443812-443279 | conserved hypothetical protein |
| BCI_0448 |  | 462970-462023 | conserved hypothetical protein |
| BCI_0478 |  | 492043-491810 | conserved hypothetical protein |
| BCI_0480 |  | 493689-493282 | conserved hypothetical protein TIGR00250 , authentic frameshift |
| BCI_0481 |  | 494252-493689 | conserved hypothetical protein |
| BCI_0516 |  | 533134-533655 | conserved hypothetical protein |
| BCI_0567 |  | 598737-598408 | conserved hypothetical protein |
| Hypothetical protein;Other | | | |
| BCI_0084 |  | 99098-99000 | hypothetical protein |
| BCI_0129 |  | 143132-143037 | hypothetical protein |
| BCI_0200 |  | 212405-212530 | hypothetical protein |
| BCI_0437 |  | 450721-450822 | hypothetical protein |
| BCI_0582 |  | 610767-611033 | hypothetical protein |
