# Supplementary material for: Metabolic Complementarity and Genomics of the Dual Bacterial Symbiosis of Sharpshooters
Source: PLoS Biol. 2006 Jun 6;4(6):e188. doi: 10.1371/journal.pbio.0040188 (PMC1472245; doi:10.1371/journal.pbio.0040188)
Supplement: Supplementary file 2 [file pbio.0040188.st002.doc]

Supplemental Table 2: Predicted protein coding genes in the *Sulcia muelleri* genome. Predicted functions and role categories are shown.

| **Accession** | **Gene**  **Symbol** | **Gene Name** |
| --- | --- | --- |
| Amino acid metabolism | | |
| SMU_011 | pruA | delta-1-pyrroline-5-carboxylate dehydrogenase |
| Amino acid biosynthesis;Aromatic amino acid family | | |
| SMU_071 | aroA | 3-phosphoshikimate 1-carboxyvinyltransferase |
| SMU_124 | aroB | 3-dehydroquinate synthase |
| SMU_170 | aroC | chorismate synthase |
| SMU_087 | aroD | 3-dehydroquinate dehydratase |
| SMU_122 | aroK | shikimate kinase |
| SMU_093 | trpE | anthranilate synthase component I |
| Amino acid biosynthesis;Aspartate family | | |
| SMU_073 | asd | aspartate-semialdehyde dehydrogenase |
| SMU_176 | aspB | aspartate Aminotransferase |
| SMU_080 | aspC | aspartate aminotransferase |
| SMU_079 | dapF | diaminopimelate epimerase |
| SMU_037 | lysA | diaminopimelate decarboxylase |
| SMU_179 | lysC | aspartokinase/homoserine dehydrogenase |
| SMU_033 | thrA | aspartokinase/homoserine dehydrogenase |
| SMU_036 | thrA | aspartokinase/homoserine dehydrogenase |
| SMU_161 | thrB | homoserine kinase |
| SMU_162 | thrC | threonine synthase |
| Amino acid biosynthesis;Glutamate family | | |
| SMU_120 | argB | acetylglutamate kinase |
| SMU_121 | argE | acetylornithine deacetylase |
| SMU_119 | argF | ornithine carbamoyltransferase |
| SMU_039 | argH | argininosuccinate lyase (partial 1) |
| SMU_171 | argH | argininosuccinate lyase (partial 2) |
| Amino acid biosynthesis;Pyruvate family | | |
| SMU_116 | ilvA | threonine dehydratase |
| SMU_113 | ilvB | acetolactate synthase large subunit |
| SMU_026 | ilvC | ketol-acid reductoisomerase |
| SMU_115 | ilvC | ketol-acid reductoisomerase |
| SMU_181 | ilvC | ketol-acid reductoisomerase |
| SMU_180 | ilvC | ketol-acid reductoisomerase (pseudogene) |
| SMU_112 | ilvD | Dihydroxy-acid dehydratase |
| SMU_114 | ilvN | acetolactate synthase small subunit |
| SMU_054 | leuA | 2-isopropylmalate synthase |
| SMU_057 | leuB | 3-isopropylmalate dehydrogenase |
| SMU_055 | leuC | 3-isopropylmalate dehydratase large subunit |
| SMU_056 | leuD | 3-isopropylmalate dehydratase small subunit |
| Amino acid biosynthesis;Serine family | | |
| SMU_084 | glyA | serine hydroxymethyltransferase |
| SMU_092 | iscS | cysteine desulfurase/cysteine sulfinate desulfinase |
| Biosynthesis of cofactors, prosthetic groups, and carriers;Lipoate | | |
| SMU_110 | lipB | lipoate-protein ligase B |
| Biosynthesis of cofactors, prosthetic groups, and carriers;Menaquinone and ubiquinone | | |
| SMU_049 | ispB | polyprenyl synthetase ispB |
| SMU_098 | ubiE | ubiquinone/menaquinone biosynthesis methyltransferase |
| Biosynthesis of cofactors, prosthetic groups, and carriers;Other | | |
| SMU_050 | sufE | cysteine desulfurase SufE subunit |
| Cell envelope;Biosynthesis and degradation of surface polysaccharides and lipopolysaccharides | | |
| SMU_048 |  | Cell wall-associated hydrolase |
| SMU_085 | rfbA | glucose-1-phosphate thymidylyltransferase putative |
| Cell envelope;Other | | |
| SMU_078 | mraW | S-adenosyl-methyltransferase MraW |
| Cellular processes;Adaptations to atypical conditions | | |
| SMU_058 | terC | integral membrane protein TerC family |
| Cellular processes;Cell division | | |
| SMU_156 | gidA | glucose-inhibited division protein A (partial 1) |
| SMU_021 | gidA | glucose inhibited division protein A (partial 2) |
| DNA metabolism;DNA replication, recombination, and repair | | |
| SMU_091 | dnaE | DNA polymerase III alpha subunit |
| SMU_029 | gyrA | DNA gyrase A subunit |
| SMU_125 | gyrA | DNA gyrase A subunit |
| SMU_083 | gyrB | DNA gyrase B subunit (partial 1) |
| SMU_007 | gyrB | DNA gyrase B subunit (partial 2) |
| SMU_003 | mutS | DNA mismatch repair protein mutS |
| SMU_022 | mutS | DNA mismatch repair protein mutS (partial 1) |
| SMU_031 | mutS | DNA mismatch repair protein mutS (partial 2) |
| Energy metabolism;ATP-proton motive force interconversion | | |
| SMU_012 |  | ATP synthase CF1 epsilon chain |
| SMU_103 | atpA | ATP synthase alpha chain |
| SMU_169 | atpB | ATP synthase beta chain (partial 1) |
| SMU_020 | atpB | ATP synthase beta chain (partial 2) |
| SMU_104 | atpE | ATP synthase C chain |
| SMU_105 | atpF | ATP synthase F0 B subunit |
| Energy metabolism;Glycolysis/gluconeogenesis | | |
| SMU_060 | gapA | glyceraldehyde 3-phosphate dehydrogenase |
| SMU_109 | maeB | NADP-dependent malate dehydrogenase (partial 1) |
| SMU_173 | maeB | NADP-dependent malic enzyme (partial 2) |
| Energy metabolism;Pyruvate dehydrogenase | | |
| SMU_172 | pdhA | Pyruvate dehydrogenase E1 component alpha subunit (partial 1) |
| SMU_002 | pdhA | Pyruvate dehydrogenase E1 component alpha subunit (partial 2) |
| SMU_082 | pdhB | pyruvate dehydrogenase E1 component beta subunit |
| SMU_059 | pdhC | Dihydrolipoamide acyltransferase E2 component |
| SMU_154 | pdhD | dihydrolipoamide dehydrogenase EC 1.8.1.4 |
| Energy metabolism;TCA cycle | | |
| SMU_075 | korA | 2-oxoglutarate ferredoxin oxidoreductase alpha subunit |
| SMU_163 | korB | 2-oxoglutarate ferredoxin oxidoreductase beta subunit |
| SMU_152 | sucA | 2-oxoglutarate dehydrogenase (decarboxylase component) |
| SMU_006 | sucB | 2-oxoglutarate dehydrogenase E2 component |
| SMU_051 | sucC | succinyl-CoA synthetase beta subunit |
| SMU_009 | sucD | succinyl-CoA synthase alpha subunit |
| Fatty acid and phospholipid metabolism;Biosynthesis | | |
| SMU_088 | acpP | acyl carrier protein |
| SMU_101 | acpP | acyl carrier protein |
| SMU_089 | fabF | 3-oxoacyl- acyl-carrier-protein synthase II |
| SMU_102 | fabF | 3-oxoacyl- acyl-carrier-protein synthase II |
| SMU_177 | ispB | polyprenyl synthetase ispB |
| Protein fate;Degradation of proteins, peptides, and glycopeptides | | |
| SMU_076 |  | CAAX amino terminal protease family protein |
| SMU_052 | clpP | ATP-dependent Clp protease proteolytic subunit 2 |
| SMU_024 | ftsH | ATP-dependent metalloprotease FtsH putative |
| SMU_040 | lon | ATP-dependent protease |
| Protein fate;Protein and peptide secretion and trafficking | | |
| SMU_016 | lspA | lipoprotein signal peptidase |
| SMU_023 | secA | preprotein translocase SecA subunit |
| SMU_146 | secY | preprotein translocase SecY subunit |
| SMU_047 | tatA | putative Sec-independent protein translocase |
| SMU_069 | tatC | putative Sec-independent protein translocase |
| Protein fate;Protein folding and stabilization | | |
| SMU_062 | dnaK | dnaK protein |
| SMU_027 | groEL | 60 kDa chaperonin GroEL |
| SMU_067 | groEL | chaperonin 60 kDa |
| SMU_005 | groES | 10 kDa chaperonin (groES) |
| SMU_019 | htpG | chaperone protein HtpG |
| Protein fate;Protein modification and repair | | |
| SMU_004 | def | polypeptide deformylase |
| SMU_160 | lgt | prolipoprotein diacylglyceryl transferase |
| Protein synthesis;Ribosomal proteins: synthesis and modification | | |
| SMU_127 | rplA | ribosomal protein L1 |
| SMU_044 | rplA | 50S ribosomal protein L1 |
| SMU_132 | rplB | 50S ribosomal protein L2 |
| SMU_130 | rplC | ribosomal protein L3 |
| SMU_131 | rplD | 50S ribosomal protein L4 |
| SMU_139 | rplE | ribosomal protein L5 |
| SMU_142 | rplF | ribosomal protein L6 |
| SMU_030 | rplK | 50S ribosomal protein L11 |
| SMU_043 | rplK | 50S ribosomal protein L11 |
| SMU_045 | rplL | ribosomal protein L7/L12 |
| SMU_138 | rplN | 50S ribosomal protein L14 |
| SMU_145 | rplO | 50S ribosomal protein L15 |
| SMU_136 | rplP | Ribosomal protein L16/L10E |
| SMU_077 | rplQ | 50S ribosomal protein L17 |
| SMU_143 | rplR | 50S ribosomal protein L18 |
| SMU_064 | rplT | ribosomal protein L20 |
| SMU_134 | rplV | 50S ribosomal protein L22 |
| SMU_159 | rplY | ribosomal protein L25 |
| SMU_165 | rpmA | 50S ribosomal protein L27 |
| SMU_096 | rpmE | ribosomal protein L31 |
| SMU_081 | rpmF | 50S ribosomal protein L32 |
| SMU_063 | rpmI | ribosomal protein L35 |
| SMU_066 | rpsB | ribosomal protein S2 |
| SMU_135 | rpsC | 30S ribosomal protein S3 |
| SMU_150 | rpsD | 30S ribosomal protein S4 |
| SMU_144 | rpsE | 30S ribosomal protein S5 |
| SMU_166 | rpsF | 30S ribosomal protein S6 |
| SMU_032 | rpsG | ribosomal protein S7 |
| SMU_141 | rpsH | ribosomal protein S8 |
| SMU_065 | rpsI | ribosomal protein S9 |
| SMU_129 | rpsJ | ribosomal protein S10 |
| SMU_149 | rpsK | ribosomal protein S11 |
| SMU_097 | rpsL | ribosomal protein S12 |
| SMU_148 | rpsM | 30S ribosomal protein S13 |
| SMU_140 | rpsN | ribosomal protein S14 |
| SMU_099 | rpsO | ribosomal protein S15 |
| SMU_126 | rpsP | 30S ribosomal protein S16 |
| SMU_070 | rpsP | 30S ribosomal protein S16 |
| SMU_137 | rpsQ | Ribosomal protein S17 |
| SMU_167 | rpsR | ribosomal protein S18 |
| SMU_133 | rpsS | 30S ribosomal protein S19 |
| SMU_072 | rpsT | ribosomal protein S20 |
| Protein synthesis;Translation factors | | |
| SMU_128 | fusA | translation elongation factor G |
| SMU_147 | infA | translation initiation factor IF-1 |
| SMU_086 | prfA | peptide chain release factor 1 |
| SMU_042 | tuf | translation elongation factor Tu |
| Protein synthesis;tRNA aminoacylation | | |
| SMU_107 | glnS | glutaminyl-tRNA synthetase (partial 1) |
| SMU_035 | glnS | glutaminyl-tRNA synthetase (partial 2) |
| SMU_034 | leuS | leucyl-tRNA synthetase |
| SMU_001 | pheS | phenylalanyl-tRNA synthetase alpha chain |
| SMU_117 | pheS | phenylalanyl-tRNA synthetase alpha chain |
| SMU_028 | pheT | phenylalanyl-tRNA synthetase beta chain |
| SMU_164 | serS | seryl-tRNA synthetase |
| SMU_008 | trpS | Tryptophanyl-tRNA synthetase |
| SMU_053 | tyrS | tyrosyl-tRNA synthetase (partial 1) |
| SMU_108 | tyrS | tyrosyl-tRNA synthetase (partial 2) |
| SMU_118 | valS | valyl-tRNA synthetase (partial 1) |
| SMU_111 | valS | valyl-tRNA synthetase (partial 2) |
| Protein synthesis;tRNA and rRNA base modification | | |
| SMU_168 | ksgA | dimethyladenosine transferase |
| SMU_038 | miaB | tRNA-i(6)A37 thiotransferase enzyme MiaB |
| SMU_094 | rluD | ribosomal large subunit pseudouridine synthase D |
| SMU_013 | trmE | tRNA modification GTPase TrmE |
| SMU_061 | trmH | tRNA/rRNA methyltransferase |
| SMU_095 | truA | tRNA pseudouridine synthase A |
| SMU_178 | truA | tRNA pseudouridine synthase A |
| Purines, pyrimidines, nucleosides, and nucleotides;Purine ribonucleotide biosynthesis | | |
| SMU_158 | prsA | ribose-phosphate pyrophosphokinase |
| Purines, pyrimidines, nucleosides, and nucleotides;Pyrimidine ribonucleotide biosynthesis | | |
| SMU_025 | carB | carbamoyl-phosphate synthase large subunit |
| Transcription;DNA-dependent RNA polymerase | | |
| SMU_151 | rpoA | DNA-directed RNA polymerase alpha subunit (partial 1) |
| SMU_010 | rpoA | DNA-directed RNA polymerase alpha chain (partial 2) |
| SMU_046 | rpoB | DNA-directed RNA polymerase beta chain (partial 1) |
| SMU_015 | rpoB | DNA-directed RNA polymerase beta subunit (partial 2) |
| SMU_100 | rpoC | DNA-directed RNA polymerase beta' chain (partial 1) |
| SMU_014 | rpoC | DNA-directed RNA polymerase beta' subunit (partial 2) |
| SMU_041 | rpoD | RNA polymerase sigma factor rpoD (Sigma-A) |
| Transcription;Degradation of RNA | | |
| SMU_017 | pnpA | polyribonucleotide nucleotidyltransferase |
| Transcription;Transcription factors | | |
| SMU_090 | rho | transcription termination factor rho |
| Unknown function | | |
| SMU_123 |  | ATP-binding PP-loop protein |
| SMU_155 | obg | GTP-binding protein Obg |
| SMU_153 |  | hypothetical protein |
| SMU_182 |  | hypothetical protein |
| SMU_018 |  | Hypothetical protein |
| SMU_175 |  | hypothetical protein FLJ35424 (frame 1) |
| SMU_157 |  | methyltransferase |
| SMU_074 |  | Mrp/Nbp35 family ATP-binding protein |
| SMU_068 |  | SsrA-binding protein |
